# Targeting the ANXA8–SP1–PPA1 Axis to Modulate TCA Cycle and Matrix Deposition in Diffuse-Type Gastric Cancer

**DOI:** 10.34133/research.0838

**Published:** 2025-08-25

**Authors:** Yuxia Wu, Xiangyan Jiang, Huiguo Qing, Yansong Hou, Yong Ma, Tao Wang, Keshen Wang, Long Qin, Weiwen Cai, Zongrui Xing, Bin Zhao, Qichen He, Wenbo Liu, Tian Wang, Haonan Sun, Xingshuo Zhao, Zuoyi Jiao, Zeyuan Yu

**Affiliations:** ^1^ Department of General Surgery, Lanzhou University Second Hospital, Lanzhou, Gansu, China.; ^2^ The Second School of Clinical Medicine, Lanzhou University, Lanzhou 730000, China.; ^3^ Cuiying Biomedical Research Center, Lanzhou University Second Hospital, Lanzhou 730030, China.; ^4^ Gansu Province High-Altitude High-Incidence Cancer Biobank, Lanzhou University Second Hospital, Lanzhou 730030, China.; ^5^ Gansu Tumor Immunology Basic Disciplines Research Center, The Second Hospital & Clinical Medical School, Lanzhou University, Gansu 730030, China.

## Abstract

Diffuse-type gastric cancer (DGC) is an aggressive tumor type characterized by a dense extracellular matrix (ECM). Metabolic reprogramming, a key oncogenic factor driving tumor progression, is closely linked to ECM deposition, although the regulatory mechanisms remain poorly understood. In this study, we integrated single-cell sequencing, proteomics, metabolomics, and large-scale clinical data to identify the metabolic signature of DGC. We found that the tricarboxylic acid (TCA) cycle is suppressed in DGC, which correlates with the formation of a dense ECM. Annexin A8 (ANXA8) was identified as a critical regulator that inhibits the TCA cycle in DGC and is positively associated with matrix formation. Mechanistically, ANXA8 interacts with SP1 to promote the transcription of pyrophosphatase 1, thereby suppressing the TCA cycle, activating cancer-associated fibroblasts, and facilitating aberrant ECM deposition. Deletion of ANXA8 suppresses malignant phenotypes and shows synergistic effects with the chemotherapeutic agent 5-fluorouracil (5-FU). Large-scale clinical data further confirmed the correlation between ANXA8 expression and both gastric cancer progression and 5-FU therapeutic efficacy. High-throughput organoid screening identified UNC2025 as a selective ANXA8 inhibitor. Targeting ANXA8 with UNC2025 restores TCA cycle activity and inhibits ECM deposition in DGC, enhancing the therapeutic effects of 5-FU in patient-derived xenografts and organoids. Furthermore, a polyphenol-based UNC2025 nanodelivery system improved the efficacy of this combination therapy. In summary, this study elucidates how ANXA8-mediated suppression of the TCA cycle promotes dense ECM formation and malignant progression in DGC, highlighting the therapeutic potential of targeting ANXA8 in DGC treatment.

## Introduction

According to the latest data from the World Health Organization’s International Agency for Research on Cancer, gastric cancer (GC) ranks fifth globally in both incidence and mortality among malignant tumors [1]. Diffuse-type gastric cancer (DGC), classified under Lauren’s classification, accounts for 30% to 50% of all GC cases. It is characterized by early onset, poor prognosis, rapid progression, and a familial predisposition [2–4]. Although recent advancements in immunotherapy and the identification of novel molecular targets have led to substantial breakthroughs in DGC treatment [5–7], their efficacy remains suboptimal [8,9]. 5-Fluorouracil (5-FU)-based chemotherapy continues to be the cornerstone of treatment for GC [10]; however, its efficacy is notably reduced in DGC compared to intestinal-type gastric cancer (IGC) [11,12], with the growing prevalence of drug resistance further impeding its therapeutic potential [13]. Histologically, DGC is classified as a poorly differentiated carcinoma, characterized by solitary or small clusters of cancer cells that infiltrate adjacent tissues with high invasiveness and a pronounced stromal response [14,15]. A central feature of extracellular matrix (ECM) remodeling in DGC is abnormal matrix deposition, driven by the activation of cancer-associated fibroblasts (CAFs) [16,17]. Cancer cells promote tumor progression by recruiting and reprogramming non-cancerous host cells, which, in turn, remodel the ECM [18]. This abnormal ECM deposition leads to hypoxia in the TME, affecting drug delivery and contributing to tumor resistance [19]. Additionally, ECM alterations impact immune cell aggregation and activation, promoting tumorigenesis and drug resistance [20–22]. Therefore, elucidating the mechanisms underlying aberrant ECM deposition in DGC is crucial for improving therapeutic outcomes and developing novel treatment strategies.

Metabolic reprogramming, a hallmark of cancer [23,24], supports cancer cell growth and proliferation by facilitating intercellular communication [25]. Metabolic alterations typical of tumors, particularly excessive lactate production, contribute to increased tumor invasion and metastasis [26]. Lactate not only stimulates the proliferation and migration of CAFs but also enhances collagen synthesis, contributing to the formation of a dense stromal microenvironment [27]. Lactate serves as a critical substrate for the tricarboxylic acid (TCA) cycle, where it is converted to pyruvate and subsequently oxidized in the mitochondria [28,29]. The TCA cycle is essential for cellular oxidative phosphorylation, fulfilling bioenergetic, biosynthetic, and redox homeostasis requirements. In the TME, the lactate–pyruvate cycle helps maintain cellular energy stability, while lactate accumulation can impair TCA cycle function, thereby affecting tumor progression [30]. Inhibition of the TCA cycle results in an inadequate energy supply, forcing cells to rely on alternative metabolic pathways, which leads to the accumulation of substrates and products. This accumulation disrupts metabolic homeostasis and impairs cellular function and survival [31–33]. TCA cycle activity influences matrix deposition through CAF-mediated synthesis and secretion of matrix components in response to lactate [27]. Therefore, the balance between the TCA cycle and lactate is crucial for both tumor metabolism and the TME. However, the role of the TCA cycle in the stromal microenvironment of DGC remains unclear and may offer potential avenues for improving the prognosis of patients with DGC.

In this study, we reveal that deficiencies in the TCA cycle are linked to aberrant ECM deposition in DGC. Mechanistically, ANXA8 interacts with SP1 to enhance pyrophosphatase 1 (PPA1) transcription, thereby inhibiting the TCA cycle and triggering CAF activation and matrix deposition. Knockout and pharmacological inhibition of ANXA8 reduce DGC progression and enhance the effects of 5-FU. Furthermore, a nanodelivery system improves the efficacy of anti-ANXA8 therapy. Overall, this study uncovers a metabolic regulatory mechanism for aberrant matrix deposition in DGC and proposes a promising therapeutic strategy.

## Results

### TCA cycle suppression correlates with aberrant ECM deposition

**Fig. 1.**
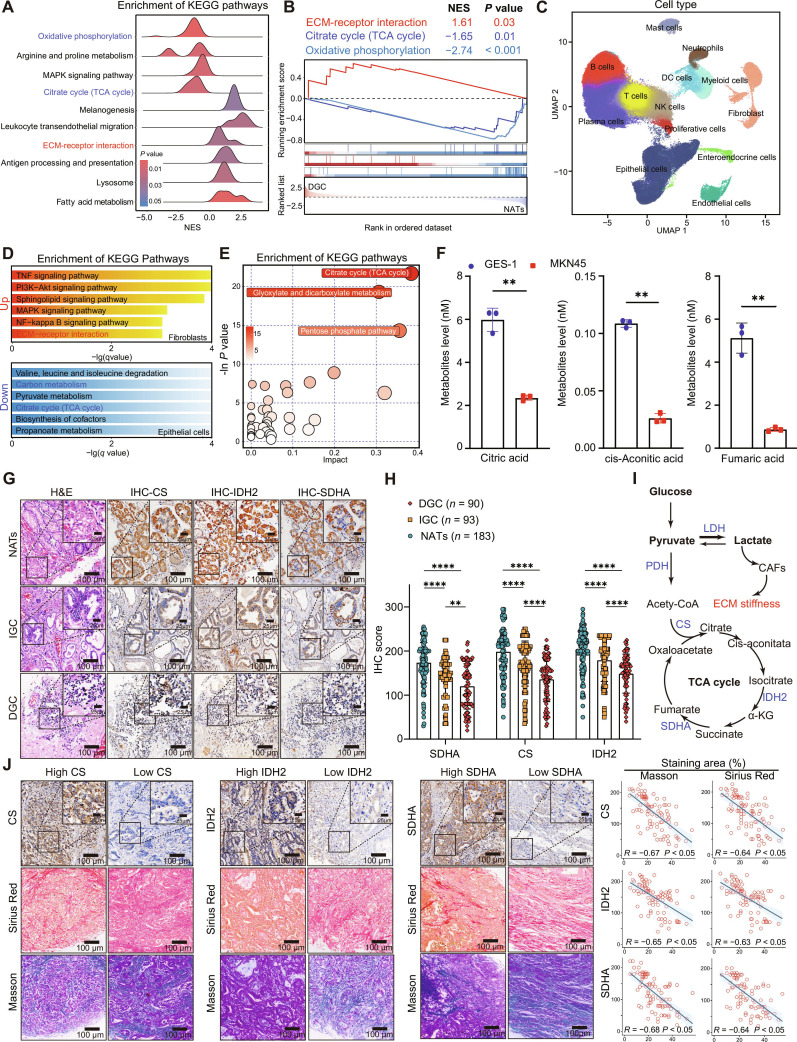
TCA cycle suppression correlates with aberrant ECM deposition. (A and B) KEGG functional enrichment analyses of differentially expressed proteins between patient-derived tumor and paired normal adjacent tissues (NATs), and enrichment maps of ECM–receptor interaction, TCA cycle, and oxidative phosphorylation pathways based on GSEA, with a 25% false discovery rate (FDR) and *P* < 0.05. (C) UMAP plots of scRNA-seq profiles, color-coded for cell types. (D) KEGG functional enrichment analyses of differentially expressed scRNA-seq profiles between patient-derived tumor tissues and paired NATs or normal tissues. (E) KEGG functional enrichment analysis of differentially expressed metabolites. (F) Quantitative statistics of citric, cis-aconitic, and fumaric acids. (G) Representative H&E and IHC staining for CS, IDH2, and SDHA in the indicated groups. Scale bar, 100 and 25 μm. (H) Quantification of CS, IDH2, and SDHA staining in NATs (*n* = 183), IGC (*n* = 93), and DGC (*n* = 90). (I) Map of energy metabolism pathways in DGC. (J) Representative images of CS, IDH2, and SDHA IHC staining, with corresponding Masson’s and Sirius Red staining in patients with DGC, and correlation analysis between each group. The group includes patients with Low CS (*n* = 49), High CS (*n* = 41), Low IDH2 (*n* = 35), High IDH2 (*n* = 55), Low SDHA (*n* = 62), and High SDHA (*n* = 28) levels. Scale bar, 100 and 25 μm. Correlations were calculated using Spearman’s correlation coefficient, and the fitted lines represent the linear regression models. Results are presented as mean ± standard deviation in (F) and (H). *n*: Biological duplication per group. Student’s *t* test, **P < 0.01; ****P < 0.0001. NES, normalized enrichment score; IGC, intestinal-type gastric cancer; KEGG, Kyoto Encyclopedia of Genes and Genomes; ECM, extracellular matrix.

We collected tumor tissues and normal adjacent tissues (NATs) from 15 patients diagnosed with DGC and applied a label-free quantitative proteomic approach to identify differentially expressed proteins. Kyoto Encyclopedia of Genes and Genomes (KEGG) enrichment analysis revealed marked suppression of the TCA cycle and oxidative phosphorylation pathways in DGC tissues compared to NATs (Fig. 1A and B and Fig. S1A). Gene Ontology (GO) enrichment analysis further supported this finding, highlighting the down-regulation of genes associated with oxidative phosphorylation and aerobic respiration pathways (Fig. S1B to F).

Additionally, we performed single-cell RNA sequencing (scRNA-seq) on primary GC samples. After quality control and data integration, a total of 261,807 single cells were analyzed, revealing 13 distinct cell populations (Fig. 1C and Fig. S2B). To explore tumor-specific metabolic alterations, paired NATs from the same patients were used as internal controls to minimize inter-individual differences. KEGG enrichment analysis of epithelial cells showed marked suppression of carbon metabolism and TCA cycling in DGC (Fig. 1D). In the ECM analysis, the focus was on fibroblasts. We identified elevated levels of malignant ECM markers (ACTA2, FAP, MMP11, INHBA, POSTN, TNC, SERPINE1, and LOX) in NATs. Consequently, normal tissues were used as controls for this analysis (Fig. S2D). The enrichment analysis revealed marked up-regulation of the ECM receptor interaction pathway in DGC. Similar changes were observed in the IGC, although they were not statistically significant in The Cancer Genome Atlas (TCGA) dataset (Fig. S2A and C).

To investigate metabolic alterations further, we performed a metabolomic analysis on GES-1 cells (derived from normal human gastric mucosal epithelial cells) and MKN45 cells (derived from human DGC tissue), which exhibit highly invasive and metastatic characteristics typical of DGC. Pathway enrichment analysis showed marked down-regulation of TCA cycle-related metabolites, including citric acid, cis-aconitic acid, and fumaric acid, in MKN45 cells (Fig. 1E and F and Fig. S3A and B). We also analyzed clinical specimens from 183 patients with GC, including 93 IGC and 90 DGC cases. Immunohistochemical (IHC) staining demonstrated reduced expression of key TCA cycle enzymes, including citrate synthase (CS), isocitrate dehydrogenase 2 (IDH2), and succinate dehydrogenase complex subunit A (SDHA), in both IGC and DGC cases (Fig. 1G and H). The proteomics analysis further revealed marked enrichment of ECM-related pathways, which aligns with the dense matrix characteristic of DGC (Fig. 1A and B). Masson’s and Sirius Red assays showed a negative correlation between the expression of TCA cycle enzymes and collagen levels (Fig. 1J). These findings suggest that the suppression of TCA cycle activity in DGC is inversely associated with collagen deposition, indicating a potential relationship between reduced TCA activity and ECM deposition in DGC (Fig. 1I).

### ANXA8 as a key regulator of TCA cycle suppression and aberrant ECM deposition in DGC

**Fig. 2.**
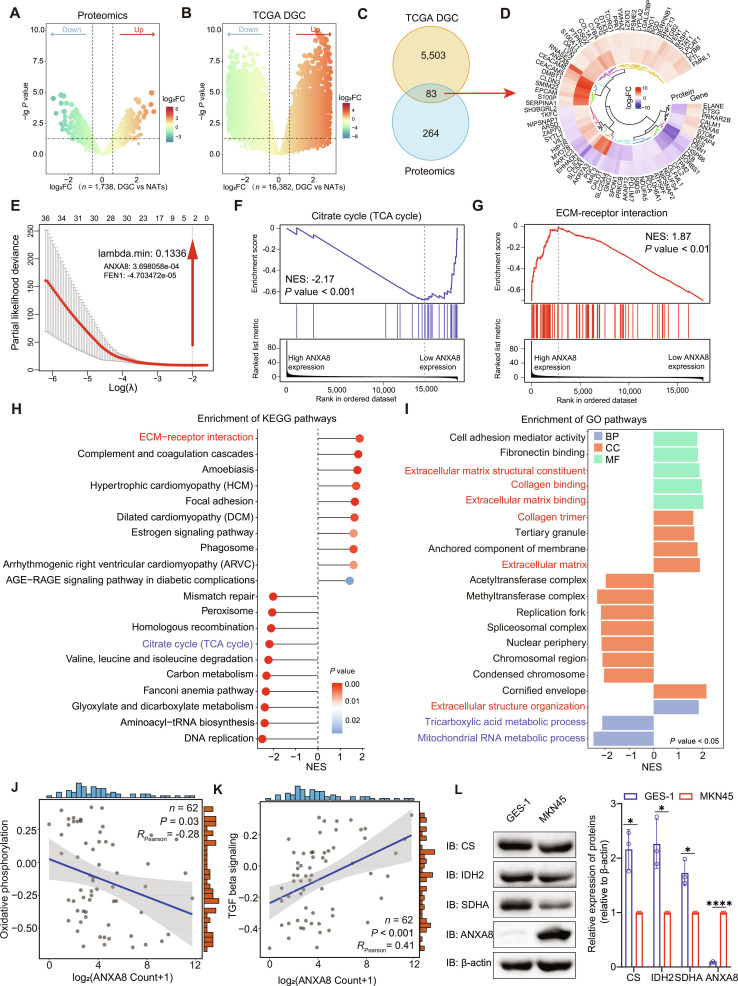
ANXA8 as a key regulator of TCA cycle suppression and aberrant ECM deposition in DGC. (A) The volcano plot delineates proteomic data comparing DGC with paired normal adjacent tissues (NATs). (B) A corresponding volcano plot illustrates TCGA mRNA data comparing DGC with NATs. (C and D) Venn diagrams and concentric heatmaps reveal the overlap between proteomics and TCGA mRNA data. (E) Partial likelihood deviance from LASSO regression, shown with the Log(λ) curve, identifies 2 mRNAs selected through LASSO Cox regression, marked by optimal values determined using the minimum criteria. (F to I) Enrichment maps of TCA cycle and ECM–receptor interaction pathways based on GSEA in LinkedOmics data, KEGG, and GO pathway analyses of ANXA8-associated genes based on GSEA in LinkedOmics data, with a 25% FDR and *P* < 0.05. (J and K) ssGSEA explores the Pearson correlation between ANXA8 and oxidative phosphorylation, and TGF-beta signaling; a fitted line represents the linear regression model. (L) Immunoblotting assay and quantitative statistics showing protein levels of CS, IDH2, SDHA, ANXA8, and β-actin in GES-1 and MKN45 cells. Results are presented as mean ± standard deviation in (L). *n*: Biological duplication per group. Student’s *t* test, *P < 0.05, ****P < 0.0001. TCGA, The Cancer Genome Atlas.

To identify potential factors associated with TCA cycle suppression, we first analyzed differentially expressed proteins (*P* < 0.05, |log_2_FC| > 1) from proteomics data (Fig. 2A) and differentially expressed genes (*P* < 0.05, |log_2_FC| > 1) from TCGA transcriptomics data (Fig. 2B). The intersection of these datasets yielded 83 differentially expressed genes (Fig. 2C and D). We then integrated TCGA transcriptomic data with survival information, removing missing values and standardizing the gene expression data to ensure data quality and comparability. To further refine the search for key genes, we performed Lasso regression analysis and optimized the regularization parameter λ (λ = 0.1336) using 10-fold cross-validation. This analysis identified 2 genes, ANXA8 and FEN1, with significant regression coefficients (ANXA8: 3.70E−04, FEN1: −4.70E−05) (Fig. 2E and Fig. S4A). These results suggest a strong correlation between ANXA8 and FEN1 with the target variable. GO enrichment analysis indicated that high ANXA8 expression was negatively correlated with both the TCA cycle and oxidative phosphorylation pathways, while it was positively correlated with ECM-related pathways (Fig. 2G and I and Figs. S4F and H and S5A to N). Single-sample gene set enrichment analysis (ssGSEA) revealed that ANXA8 expression was inversely correlated with oxidative phosphorylation and positively correlated with transforming growth factor-beta signaling pathways (Fig. 2J and K).

At the protein level, immunoblotting (IB) assays showed reduced expression of key TCA cycle enzymes (CS, IDH2, and SDHA) in MKN45 cells compared to GES-1 cells (Fig. 2L). IHC analysis further confirmed that ANXA8 expression was elevated in DGC tissues (Fig. S6A). These data suggest that ANXA8 down-regulates the TCA cycle and promotes collagen expression, positioning it as a potential therapeutic target for modulating aberrant ECM deposition in DGC.

### ANXA8 inhibits TCA cycle and aberrant ECM deposition in DGC

**Fig. 3.**
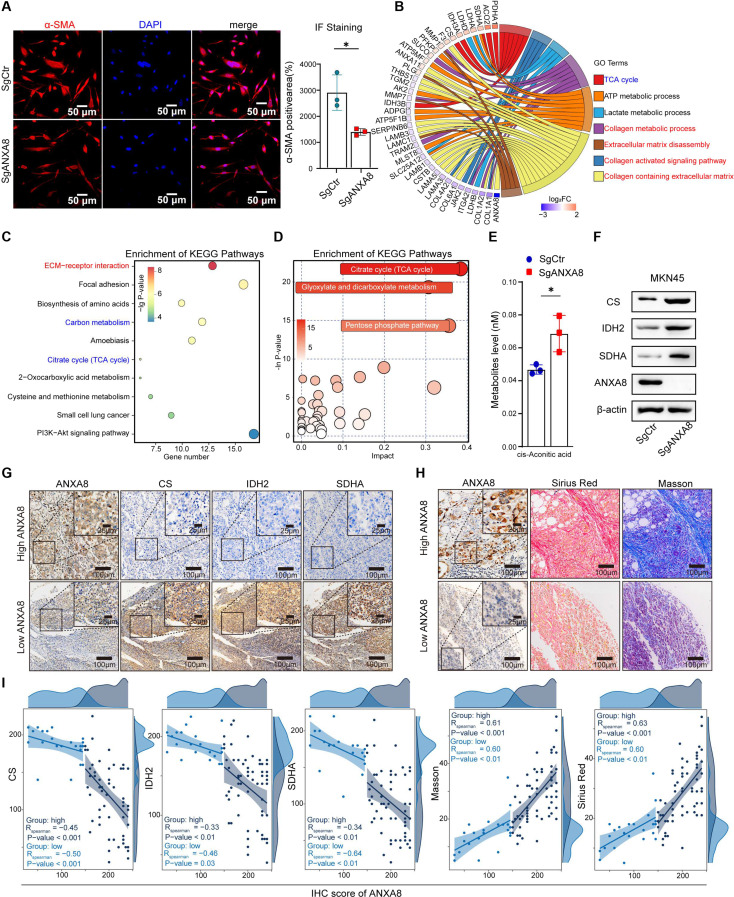
ANXA8 inhibits TCA cycle and aberrant ECM deposition in DGC. (A) Representative fluorescence microscopy images and quantitative statistics illustrating the effect of SgANXA8 on CAFs (original magnification ×20; *n* = 3). Scale bar, 50 μm. (B) GO functional enrichment analysis of differentially expressed proteins between SgANXA8 and control MKN45 cells. (C and D) KEGG pathway enrichment analysis of differentially expressed proteins and metabolites between SgANXA8 and control MKN45 cells. (E) Quantitative assessment of cis-aconitic acid levels. (F) Quantitative statistics of immunoblotting assay showing protein levels of CS, IDH2, SDHA, ANXA8, and β-actin in MKN45 cells. (G to I) Representative images and correlation analysis of IHC staining for CS, IDH2, SDHA, and Masson’s and Sirius Red staining in patients with DGC exhibiting high (IHC score ≥150, *n* = 67) versus low (IHC score <150, *n* = 23) ANXA8 expression. Scale bar, 100 and 25 μm. Quantitative statistics showed a Spearman correlation between CS, IDH2, SDHA, Masson’s, Sirius Red, and ANXA8; a fitted line represents the linear regression model. Results are presented as mean ± standard deviation in (A), (E), and (F). *n*: Biological duplication per group. Student’s *t* test, *P < 0.05. GO, Gene Ontology.

To further elucidate the role of ANXA8 in DGC, we knocked out ANXA8 (SgANXA8) in MKN45 cells. Co-culture experiments with CAFs revealed that control (Ctr) cells exhibited elevated levels of CAFs and α-smooth muscle actin expression compared to SgANXA8 cells (Fig. 3A and Fig. S6B). To assess the impact of ANXA8 on intracellular metabolism and ECM composition, we performed proteomic and metabolomic analyses. GO enrichment analysis of the differentially expressed proteins showed a marked reduction in ECM-associated functions following ANXA8 knockdown, while KEGG enrichment analysis indicated enrichment of the TCA cycle pathway in SgANXA8 cells (Fig. 3B and C and Fig. S6D and E). TCA cycle-related metabolites, including cis-aconitic acid, were up-regulated in SgANXA8 cells (Fig. 3D and E and Fig. S7A). IB assays further confirmed that ANXA8 deletion restored the expression of key TCA cycle enzymes (Fig. 3F and Figs. S6C and S7B to D).

IHC analysis of 90 DGC tissue microarray (TMA) samples demonstrated that the expression levels of TCA cycle enzymes (CS, IDH2, and SDHA) were diminished in samples with high ANXA8 expression and elevated in those with low ANXA8 expression (Fig. 3G and I). Additionally, high ANXA8 expression positively correlated with collagen deposition, and vice versa (Fig. 3H and I).

Thus, ANXA8 serves as a potential regulator of metabolic inhibition and aberrant ECM deposition in DGC.

### ANXA8 regulates the TCA cycle and ECM through interaction with PPA1 and SP1

**Fig. 4.**
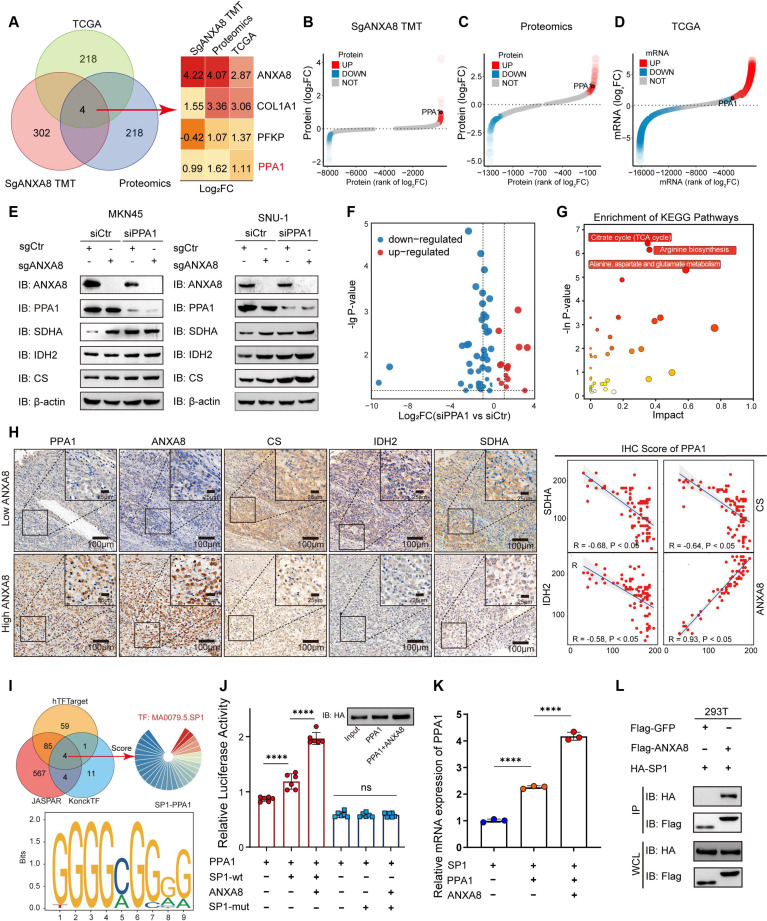
ANXA8 regulates the TCA cycle and ECM through interaction with PPA1 and SP1. (A) Venn diagrams and heatmaps show the overlap between proteomics, SgANXA8 TMT data, and TCGA mRNA data. (B) TMT data comparing SgANXA8 with SgCtr in MKN45 cells. (C) TCGA mRNA data comparing DGC with NATs. (D) TCGA data comparing DGC with NATs. (E) Immunoblotting analysis of CS, IDH2, SDHA, ANXA8, PPA1, and β-actin in SgCtr and SgANXA8 cells, with or without siPPA1 in MKN45 and SNU-1 cells. (F and G) Differentially expressed metabolites and KEGG functional enrichment analysis. (H) Representative images and correlation analysis of IHC staining for ANXA8, CS, IDH2, and SDHA in DGC patients with high (IHC score ≥150, *n* = 58) versus low (IHC score <150, *n* = 32) PPA1 expression. Scale bar, 100 and 25 μm. Quantitative statistics reveal a Spearman correlation between ANXA8, CS, IDH2, SDHA, and PPA1, with a fitted line representing the linear regression model. (I) Venn diagrams and heatmaps reveal the overlap TF of PPA1 between hTFTarget, JASPAR, and KonckTF data. The most marked score binding with the PPA1 promoter is MA0079.5.SP1, PPA1 motif (bottom). (J) DNA pull down assay showing the interaction of SP1 with PPA1 promoter (top), and dual-luciferase gene reporter assays detecting the transcriptional activity of the indicated PPA1 promoter with or without SP1 and ANXA8 overexpression with or without SP1 mut (bottom). (K) Quantitative real-time PCR (QRT-PCR) assays were employed to detect the effects of ANXA8, and SP1 on PPA1 expression. (L) Co-immunoprecipitation (Co-IP) assays were employed to assess the interaction of ANXA8 with SP1 in 293T cells expressing the indicated plasmids, with GFP serving as a control. Results are presented as mean ± standard deviation in (E), (J), and (K). *n*: Biological duplication per group. Student’s *t* test, ****P < 0.0001, ns: not significant.

To investigate the specific mechanisms by which ANXA8 regulates the TCA cycle and ECM, we utilized multi-omics data to identify genes highly expressed in cancer tissues. Our analysis revealed that inorganic PPA1, a key initiator of cellular metabolism, is markedly up-regulated (Fig. 4A to D). As an enzyme involved in energy metabolism, PPA1 is prominently expressed in various malignant tumors and contributes to tumor progression through multiple pathways [34]. We observed that PPA1 inhibits the expression of key TCA cycle enzymes. Conversely, knockdown of either ANXA8 or PPA1 resulted in increased expression of these enzymes. Simultaneous knockdown of both PPA1 and ANXA8 led to a marked up-regulation of TCA cycle enzyme expression, suggesting that the effect of ANXA8 on TCA cycle enzymes is dependent on PPA1 (Fig. 4E and Fig. S8A to C). To explore the direct relationship between PPA1 and the TCA cycle, we conducted a comprehensive metabolomic analysis using a small interfering RNA (siRNA)-mediated PPA1 knockdown cell model. Our results revealed that, compared to the control group, the TCA pathway was markedly altered in the PPA1 knockdown group. After PPA1 knockdown, concentrations of key TCA metabolites, including citric acid, cis-aconitic acid, isocitric acid, and α-ketoglutaric acid, were elevated (Fig. 4F and Fig. S8D to J). KEGG pathway enrichment analysis further confirmed marked alterations in the TCA cycle (Fig. 4G). Additionally, following PPA1 knockdown, L-lactic acid levels decreased (Fig. S8K). These metabolic changes, along with the restoration of TCA intermediates, provide compelling evidence that PPA1 acts as an inhibitor of the TCA cycle.

IHC analysis revealed a positive correlation between high expression levels of PPA1 and ANXA8, and a negative correlation with the expression of key TCA cycle enzymes (Fig. 4H). To identify transcription factors involved in regulating PPA1, we predicted its promoter sequence-bound factors using the JASPAR, hTFTarget, and Konck TF databases (Fig. 4I). We then performed a DNA pull-down assay, which demonstrated that SP1 interacts with the PPA1 promoter (Fig. 4J). We found that SP1 promotes PPA1 transcription, and ANXA8 further enhances this transcriptional activity. However, this facilitating effect was abolished when SP1 was mutated (Fig. 4J). Additionally, the mRNA expression of PPA1 was positively correlated with both SP1 and ANXA8 expression (Fig. 4K). Using AlphaFold3 simulations, we confirmed that ANXA8 binds to SP1, an interaction that is essential for regulating ANXA8’s transcriptional activity (Fig. 4L and Fig. S8L). These results confirm that ANXA8 interacts with SP1 to promote PPA1 transcription and that mutations in SP1 disrupt this interaction.

Collectively, these findings suggest a mechanism in which ANXA8, through its interaction with SP1, enhances PPA1 transcription. This leads to the inhibition of the TCA cycle and the subsequent activation of CAFs and stromal deposition.

### ANXA8 confers DGC progression and impairs 5-FU therapeutic efficacy

**Fig. 5.**
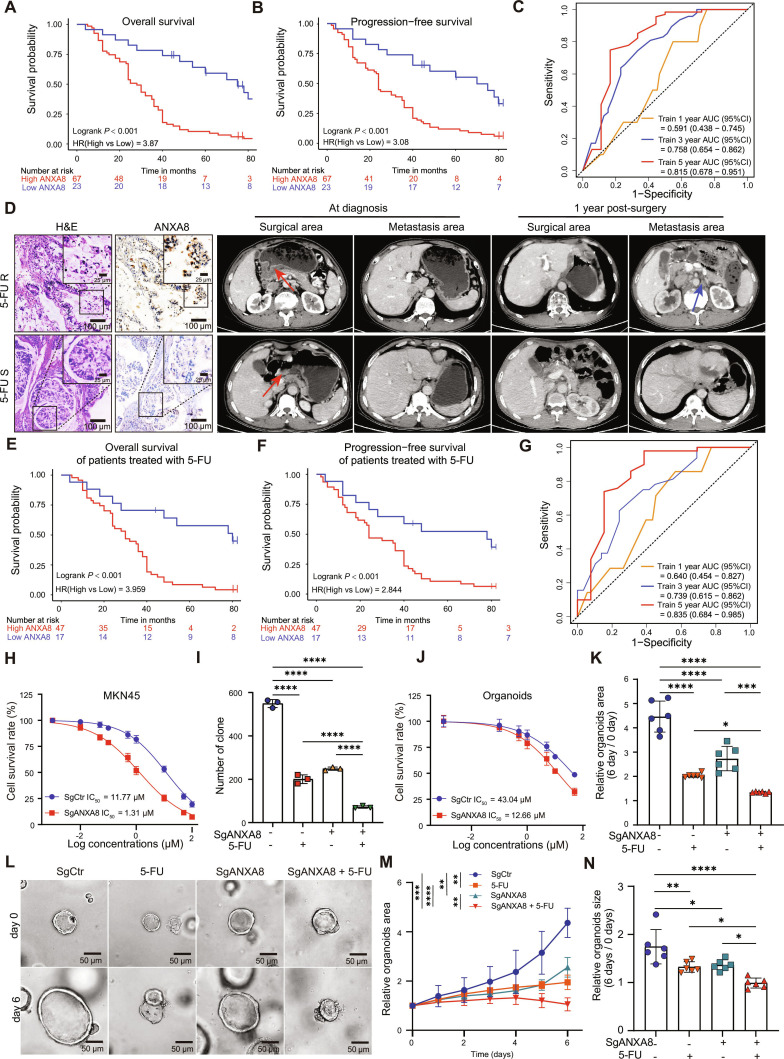
ANXA8 confers DGC progression and impairs 5-FU therapeutic efficacy. (A and B) Kaplan–Meier analysis of the correlation between ANXA8 expression (*n* = 90) and prognosis of patients with DGC in TMA samples. (C) ROC curve demonstrating the diagnostic value of ANXA8 levels in 90 DGC patients. (D) Representative H&E and IHC staining for ANXA8 and computed tomography scans in 5-FU-S (sensitive) and 5-FU-R (resistant) patients. Scale bar, 100 and 25 μm. The red arrow indicates primary tumor; the blue arrow indicates recurrent tumor. (E and F) Kaplan–Meier analysis of the correlation between ANXA8 expression (*n* = 64) and prognosis of patients subjected to 5-FU-based therapies. (G) ROC curve illustrating the diagnostic value of ANXA8 levels in distinguishing 5-FU resistance in 64 patients with DGC. (H) Sensitivity to 5-FU in control (Ctr) and SgANXA8 MKN45 cells (*n* = 3). (I) Quantification of colony formation assays assessing sensitivity to 5-FU (10 μM) in Ctr and SgANXA8 MKN45 cells (*n* = 3). (J) Sensitivity to 5-FU in Ctr and SgANXA8 PDOs (*n* = 3). (K to N) Representative images and quantification of relative area and size for the sensitivity to 5-FU (20 μM) in Ctr and SgANXA8 PDOs (*n* = 6). All data are presented as mean ± standard deviation (SD). Significance was calculated using the log-rank (Mantel Cox) test in (A), (B), (E), and (F). Statistical analysis was conducted using one-way ANOVA, followed by Tukey’s multiple comparisons post hoc test in (I), (K), and (N). Welch’s ANOVA, followed by Tamhane’s T2 multiple comparisons hoc test in (M). *n*: Biological duplication per group. *P < 0.05, **P < 0.01, ***P < 0.001, ****P < 0.0001.

We conducted a comprehensive survival analysis using clinical data from the TCGA database. Elevated ANXA8 expression was negatively correlated with overall survival (OS) (Fig. S9A). Further survival analyses of clinical samples demonstrated markedly reduced OS and progression-free survival (PFS) in the high ANXA8 expression group compared to the low expression group (Fig. 5A and B). Cox proportional hazards regression models confirmed that ANXA8 expression serves as an independent prognostic indicator of long-term survival in patients with GC (Fig. S9B and C). Additionally, receiver operating characteristic (ROC) curve analysis revealed that ANXA8 had a notable diagnostic value, with an area under the curve (AUC) of 0.815 for predicting 5-year survival (Fig. 5C).

Resistance to 5-FU, a primary chemotherapeutic agent for GC, is a major challenge in treatment [12]. We evaluated the predictive value of ANXA8 during 5-FU chemotherapy. Patients were stratified into the 5-FU-sensitive and drug-resistant groups based on computed tomography results and further categorized by ANXA8 expression levels (low: IHC score <150, high: IHC score ≥150) (Fig. 5D). Elevated ANXA8 protein levels were markedly associated with a poorer prognosis in patients undergoing 5-FU-based chemotherapy (hazard ratio [HR (High vs. Low)]: 3.959) (Fig. 5E and F). ROC curve analysis supported the diagnostic efficacy of ANXA8 in predicting 5-FU chemosensitivity (AUC: 0.835 for 5-year survival) (Fig. 5G). Validation using TCGA and GEO data confirmed that ANXA8 expression was markedly elevated in GC, particularly in stages II and III, and was associated with poor long-term survival (Fig. S9D to K). Furthermore, ANXA8 deletion in MKN45 cells markedly reduced the clonogenic and invasive abilities of these cells (Fig. S10A and b). ANXA8 deletion also enhanced the sensitivity to 5-FU of MKN45 cells and patient-derived organoid (PDO) models (Fig. 5H and J). The combination of 5-FU treatment and ANXA8 deletion resulted in a marked reduction in colony formation and organoid growth, indicating a synergistic inhibitory effect (Fig. 5I and K to N and Fig. S10C). These findings underscore the pivotal role of ANXA8 in modulating the metabolic and stromal landscapes of DGC.

In conclusion, ANXA8 is a critical biomarker for both the prognostic evaluation and prediction of chemotherapy efficacy in DGC. Deletion of ANXA8 enhances the therapeutic efficacy of 5-FU, highlighting its potential as a therapeutic target.

### Identification and characterization of UNC2025 as an ANXA8 inhibitor that modulates aberrant ECM deposition and TCA suppression

**Fig. 6.**
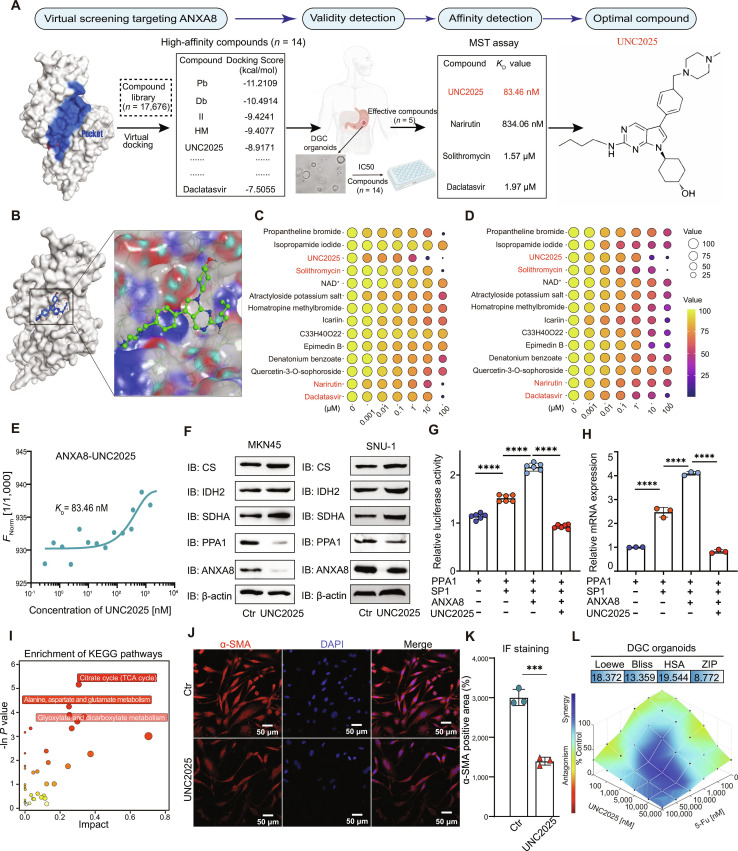
Identification and characterization of UNC2025 as an ANXA8 inhibitor. (A) Schematic representation of the screening process for ANXA8 inhibitors. (B) Computational model and interactions between UNC2025 and ANXA8. (C) Sensitivity analysis of 14 small-molecule inhibitors in DGC PDOs. (D) Sensitivity analysis of the 14 small-molecule inhibitors in MKN45 cells. (E) Determination of the kinetic constant (*K*_D_) for the interaction between UNC2025 and ANXA8 using MST. (F) Immunoblotting assay showing the protein level of CS, IDH2, SDHA, ANXA8, PPA1, and β-actin protein in Ctr and treatment (UNC2025, 5 μM) of MKN45 and SNU-1 cells. (G) Dual-luciferase gene reporter assays detecting the transcriptional activity of the indicated PPA1 promoter with or without SP1 and ANXA8 overexpression and with or without UNC2025. (H) QRT-PCR assays were employed to detect the effects of UNC2025, ANXA8, and SP1 on PPA1 expression. (I) KEGG pathway enrichment analysis of differentially expressed metabolites between Ctr and treatment (UNC2025, 5 μM) of MKN45 cells. (J and K) Representative fluorescence microscopy images and quantitative statistics illustrating the effect of UNC2025 on CAFs (original magnification ×20; *n* = 3). Scale bar, 50 μm. (L) Synergistic analysis of UNC2025 combined with 5-FU using the HSA, Loewe, and Bliss independence models in DGC PDOs. Heatmap representation of the synergistic scores of UNC2025 and 5-FU according to the ZIP, Loewe, Bliss, and HSA models. Results are presented as mean ± standard deviation in (G), (H), and (K). *n*: Biological duplication per group. Student’s *t* test, ***P < 0.001, ****P < 0.0001. Pb, propantheline bromide; Db, denatonium benzoate; II, isopropamide iodide; HM, homatropine methylbromide.

To identify small molecules targeting ANXA8, we analyzed its protein structure (Protein Data Bank ID: 1W3W) and identified 3 potential inhibitor-binding pockets (Fig. 6A and B). Using virtual screening of a library of 17,676 small molecules, we selected 14 potential compounds based on their docking scores, skeletal diversity, and molecular weights (Figs. S11 and S12). Efficacy assays in DGC PDOs and MKN45 cells led to the prioritization of 4 compounds: UNC2025, Solithromycin, Daclatasvir, and Narirutin (Fig. 6C and D). Among these, UNC2025 emerged as the most potent ANXA8 inhibitor, exhibiting a high binding affinity (*K*_D_ = 83.46 nmol/l) as determined by microscale thermophoresis (MST) (Fig. 6E and Fig. S13A to C). UNC2025 was found to inhibit the expression of ANXA8 and PPA1 while restoring the expression of key TCA cycle enzymes, including CS, IDH2, and SDHA (Fig. 6F and Fig. S13D and E).

Additionally, knockdown of PPA1 and SP1 further enhanced the cytotoxic effects of UNC2025 on MKN45 cells (Fig. S14A and B). Dual luciferase assays revealed that UNC2025 inhibited the transcription of PPA1, and it also reduced the mRNA expression of PPA1. Furthermore, UNC2025 inhibited the binding of ANXA8 to SP1 (Fig. 6G and H and Fig. S13F). After UNC2025 treatment, concentrations of key TCA metabolites, including citric acid, cis-aconitic acid, isocitric acid, and α-ketoglutaric acid, were elevated and L-lactic acid levels decreased (Fig. S13G to M). KEGG pathway enrichment analysis further confirmed marked alterations in the TCA cycle (Fig. 6I). In summary, UNC2025 restored TCA cycle activity by inhibiting ANXA8 expression, disrupting its interaction with SP1, and down-regulating PPA1 transcription.

To confirm the inhibitory effect of UNC2025 on ANXA8, we treated CAFs with UNC2025 and co-cultured them in MKN45-conditioned medium. Treatment with UNC2025 led to markedly reduced cell numbers and fluorescence intensities compared to the control group (Fig. 6J and K). To assess the synergistic potential of UNC2025 and 5-FU, we applied the Zero Interaction Potency (ZIP), Loewe, Bliss, and Highest Single Agent (HSA) models. Our analysis identified optimal synergy at 1 μM UNC2025 combined with 20 μM 5-FU in PDOs, and at 5 μM UNC2025 combined with 10 μM 5-FU in MKN45 cells (Fig. 6L and Fig. S14C to O).

Collectively, these findings highlight that UNC2025 is a selective small-molecule inhibitor of ANXA8, exhibiting potent synergism with 5-FU. This suggests its potential as a therapeutic agent for DGC.

### The combination of UNC2025 and 5-FU reduces tumor growth and improves survival in preclinical DGC models

**Fig. 7.**
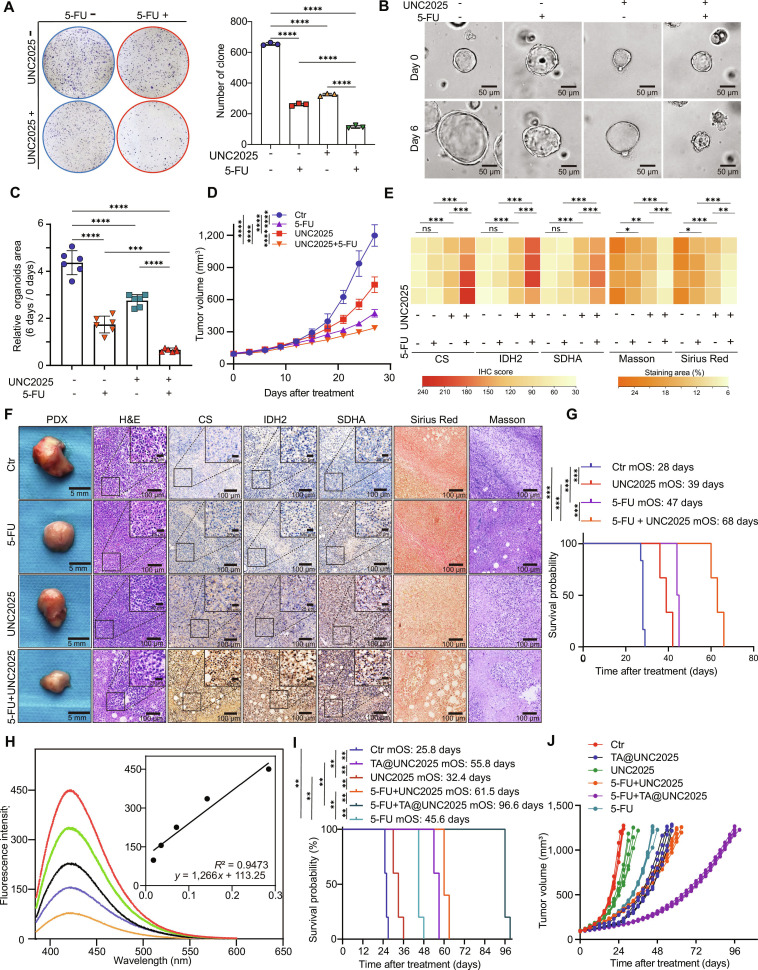
UNC2025 increases 5-FU efficacy and further enhances synergy with 5-FU after nanopackaging. (A) Representative images and quantification of colony formation assays assessing sensitivity to 5-FU (10 μM), UNC2025 (5 μM), or both (*n* = 3). (B and C) Representative images and quantification of PDOs response to UNC2025 (1 μM) and 5-FU (20 μM) (*n* = 6). Scale bar, 50 μm. (D) Tumor growth in PDXs treated with UNC2025, 5-FU, or both (*n* = 4). (E and F) Raw tumor volume, representative H&E staining, IHC staining, Masson’s and Sirius Red staining, and quantification in tumor tissues of PDXs treated with UNC2025 or/and 5-FU. Scale bar, 5 mm, 100 μm, and 25 μm. (G) Kaplan–Meier analysis of PDXs treated with UNC2025, 5-FU, or both. Significance was determined by the log-rank test. (H) Fluorescence values of different concentrations of UNC2025 and standard curve. (I) Kaplan–Meier analysis of PDX with UNC2025, TA@UNC2025, or combination therapy with 5-FU, and 5-FU alone, respectively. (J) Tumor growth of PDX with UNC2025, TA@UNC2025, or combination therapy with 5-FU, and 5-FU alone, respectively (*n* = 5). Significance was assessed using the log-rank (Mantel Cox) test in (G) and (I). All data are presented as mean ± standard deviation (SD). Statistical analysis was conducted using one-way ANOVA, followed by Tukey’s multiple comparisons post hoc test in (A), (C), and (E). Generalized estimating equations in (D). *n*: Biological duplication per group. **P < 0.01, ***P < 0.001, ****P < 0.0001, ns: not significant.

To assess the effect of ANXA8 inhibition on the efficacy of 5-FU, MKN45 cells and PDO models were treated with either 5-FU, UNC2025, or a combination of both. Both UNC2025 and 5-FU independently suppressed cell clonogenicity and invasion, and their combination further enhanced the inhibition of tumor cell colony formation and invasion (Fig. 7A and Fig. S15A and B). Similar results were observed in PDOs, where both UNC2025 and 5-FU independently inhibited organoid growth, and their combination resulted in an even greater reduction in organoid growth (Fig. 7B and C and Fig. S15F). In the presence of 5-FU, both UNC2025 treatment and SgANXA8 reduced colony formation to similar extents, and no additive effect was observed with the combination of the 2 treatments (Fig. S15C to E). This suggests that UNC2025 enhances 5-FU sensitivity through specific targeting of ANXA8.

To further investigate the potential of UNC2025 in enhancing 5-FU efficacy, we employed a patient-derived xenograft (PDX) model of DGC. UNC2025 treatment markedly suppressed tumor growth rates in the PDX model (Fig. 7D and Fig. S15G to I). Additionally, UNC2025 markedly reduced abnormal ECM deposition in the PDX tissues and restored the expression of key TCA cycle enzymes in DGC tissues (Fig. 7E and F). Furthermore, when combined with 5-FU, UNC2025 not only inhibited tumor growth more effectively than 5-FU monotherapy but also markedly prolonged survival (median OS: 68 days vs. 47 days; Fig. 7G and Fig. S15I).

These results demonstrate that ANXA8-mediated suppression of the TCA cycle and pathological ECM deposition critically drive DGC malignant progression and contribute to reduced efficacy of 5-FU therapy.

### Polyphenol nanodelivery system further enhances the efficacy of ANXA8 inhibitors

To improve the bioavailability and in vivo activity of UNC2025, we developed a nanostructure co-assembled from hyaluronic acid (HA) and tannic acid (TA). Both PEG-HA and TA exhibit excellent biocompatibility, ensuring the safety and tolerability of the nanomedicine in vivo. The hydroxyl groups of PEG-HA and the phenolic hydroxyl groups of TA form hydrogen bonds, driving the self-assembly into TA-HA nanoparticles (NPs), with an average hydrodynamic diameter of 917.29 nm and a polydispersity index (PDI) of 0.436 (Fig. S15M). Following successful loading of UNC2025 and TA-HA via hydrophobic interactions, core-shell NPs labeled as TA@UNC2025 were formed. The Tyndall effect observed in the TA@UNC2025 aqueous solution confirmed the self-assembly of the nanoparticles. Transmission electron microscopy (TEM) images revealed that the PEG-HA, TA, and UNC2025 co-assembled into uniform spherical nanostructures with distinct core–shell morphology. These structures had an average diameter of 20 nm, narrow size distribution, and well-defined boundaries, demonstrating robust self-assembly capabilities and morphological stability (Fig. S15J). Fluorescence spectrophotometry indicated a drug loading content of 30% for UNC2025 in the nanomedicine (Fig. 7H).

Dynamic light scattering (DLS) analysis showed that TA@UNC2025 exhibited a narrow size distribution with an average hydrodynamic diameter of 190.66 nm and a PDI of 0.267, indicating homogeneity and colloidal stability (Fig. S15N). The increased hydrophobicity of UNC2025 reduced the particle size, ensuring stability. Ultraviolet–visible absorption spectroscopy revealed a redshift of UNC2025’s characteristic absorption peak from 295 to 285 nm after incorporation into the nanostructure, confirming successful drug loading (Fig. S15K). Fourier-transform infrared spectroscopy (FT-IR) further confirmed the presence of characteristic functional groups in each component of TA@UNC2025 (Fig. S15L). The zeta potential of TA-HA NPs was −63.52 mV, which decreased to −50 mV after UNC2025 loading (Fig. S15O). This negative surface charge contributes to long-term stability during systemic circulation. In vitro cumulative release profiles of UNC2025 from the nanomedicine were evaluated in both pH 7.4 PBS and pH 5.0 acetate buffer (ABS) at 37 °C. The nanomedicine demonstrated pH-dependent release kinetics, with 65.86% cumulative release at pH 5.0 (simulating the tumor microenvironment) compared to only 9.58% at pH 7.4 (physiological conditions) over 36 h, confirming tumor-targeted drug release (Fig. S15P). Additionally, the toxicity of TA@UNC2025 on MKN45 cells was more than twofold higher than that of free UNC2025 (Fig. S15Q).

To assess the therapeutic efficacy of TA@UNC2025, the nanomedicine was tested in a DGC PDX model. Compared to free UNC2025, TA@UNC2025 markedly enhanced antitumor efficacy, extending the median survival of mice from 32.4 to 55.8 days (Fig. 7I and J). Compared to 5-FU, TA@UNC2025 markedly enhanced antitumor efficacy, extending the median survival of mice from 45.6 to 55.8 days. Furthermore, combination therapy with TA@UNC2025 and 5-FU prolonged median survival to 96.6 days, outperforming the 61.5 days achieved with UNC2025 plus 5-FU. These results collectively highlight the superior antitumor potency of the nanomedicine.

Biosafety was evaluated via H&E staining of major organs (heart, liver, spleen, lungs, and kidneys). After tumors reached 1,200 mm^3^, no marked histopathological alterations were observed in the liver, spleen, kidneys, or lungs across treatment groups, indicating negligible organ toxicity. However, free UNC2025 induced severe cardiotoxicity, as evidenced by H&E-stained heart sections showing disintegration and fragmentation of myocardial fibers (arrows). In contrast, no cardiac abnormalities were detected in the TA@UNC2025-treated group, likely attributable to controlled drug release, further validating the biocompatibility of the nanomedicine (Fig. S16).

## Discussion

Aberrant ECM deposition is a hallmark of DGC that contributes to tumor progression and impairs drug delivery, thereby reducing the efficacy of chemotherapy [35]. We first investigated the metabolic abnormalities in DGC and discovered a marked absence of the TCA cycle, which is closely correlated with ECM remodeling. We identified ANXA8 as a key factor involved in both TCA cycle dysregulation and aberrant ECM deposition; it suppresses the TCA cycle by interacting with SP1, thereby increasing PPA1 transcription. This process activates CAFs and promotes aberrant ECM deposition, with marked implications for DGC prognosis and malignancy. Our study is the first to demonstrate that pharmacological inhibition of ANXA8, coupled with an innovative nanomedicine delivery system, can restore TCA cycle activity, mitigate aberrant ECM deposition, and overcome chemoresistance in DGC.

The formation of ECM deposits in tumors often disrupts metabolic homeostasis, impedes intercellular communication, and exacerbates metabolic dysregulation in cancer cells [36]. Tumors with increased glycolytic activity accumulate lactic acid. Under normal conditions, lactate serves as a critical fuel for the TCA cycle [29]; however, our study revealed suppression of the TCA cycle in DGC. This phenomenon has also been observed in pancreatic ductal adenocarcinoma, non-small cell lung cancer, and colon cancer [33], in contrast to breast cancer metastasis and leukemia models, which show higher TCA flux [37]. Thus, TCA cycle activity may be related to tumor-specific histological characteristics. Inhibition of the TCA cycle reduces ATP production and leads to the accumulation of metabolite intermediates, thereby driving tumor progression [38–40]. In DGC, we have shown that TCA cycle inhibition leads to excessive lactic acid accumulation, which stimulates collagen synthesis by CAFs within the ECM, exacerbating aberrant ECM deposition.

ANXA8, a member of the calcium-dependent phospholipid-binding protein family, plays a pivotal role in various cellular functions [41]. We have shown that ANXA8 has tumor-promoting functions in DGC. Further validation by multi-omics analyses of ANXA8 knockdown in MKN45 cells confirmed its involvement in TCA cycle suppression and ECM remodeling. ANXA8 is up-regulated in several malignancies and contributes to cell proliferation and drug resistance [42–46]. Similarly, we observed increased ANXA8 expression in DGC, which enhanced malignant proliferation and invasion. Using TMAs of human DGC specimens, we found that high ANXA8 expression levels were associated with poor survival outcomes and resistance to 5-FU. Therefore, we propose that ANXA8 drives the malignant progression of DGC by orchestrating TCA cycle disruption and aberrant ECM deposition, making it a potential therapeutic target.

PPA1 is an enzyme that catalyzes the hydrolysis of pyrophosphate and is involved in many biochemical reactions and diseases progression [47,48]. Multi-omics analyses have shown that PPA1 is overexpressed in tumors, where it inhibits key enzymes in the TCA cycle. Our metabolomic analysis using an siRNA-mediated PPA1 knockdown cell model provided direct evidence of PPA1’s role in regulating the TCA cycle. Knockdown of PPA1 led to marked alterations in the TCA pathway, characterized by elevated levels of key metabolites such as citric acid, cis-aconitic acid, isocitric acid, and α-ketoglutaric acid. At the same time, we observed a decrease in L-lactic acid levels following PPA1 knockdown. These changes, together with the restoration of TCA intermediates, strongly support the conclusion that PPA1 functions as an inhibitor of the TCA cycle. Mechanistically, our study suggests that the interaction between ANXA8 and SP1 enhances PPA1 transcription, which, in turn, suppresses the TCA cycle. This metabolic shift contributes to the activation of CAFs and the deposition of stromal components. Targeting the ANXA8–SP1–PPA1 axis could, therefore, offer a promising therapeutic strategy to inhibit the progression of DGC.

To validate the therapeutic potential of targeting ANXA8, UNC2025 was identified as a selective ANXA8 inhibitor. UNC2025, a small-molecule inhibitor of the MERTK/FLT3 pathway, has demonstrated important antitumor activity against acute lymphoblastic leukemia and acute myeloid leukemia [49,50]. In addition, previous studies have highlighted the potent antitumor effects of UNC2025 in leukemia [49], gastric adenocarcinoma [51], and meningioma [52], as well as its ability to counteract chemoresistance in Ewing sarcoma [53]. Our data confirm that ANXA8 interacts with SP1 to promote PPA1 transcription, with UNC2025 markedly inhibiting this interaction. In vitro and in vivo pharmacological inhibition of ANXA8 with UNC2025 effectively restored TCA cycle activity, reduced stromal density, and attenuated DGC progression and chemoresistance. With the rapid development of materials and chemical technology, nanoparticle-mediated drug delivery systems have played a important role in improving the biocompatibility of drugs, enhancing tumor targeting and the antitumor effect of drugs, and have shown promising prospects [54,55]. To enhance the bioavailability and therapeutic efficacy of UNC2025, we developed polyphenol-based nanostructures encapsulated with HA and TA. This novel drug delivery system, which is characterized by superior biocompatibility and pH sensitivity [56,57], markedly improved the bioavailability and antitumor activity of UNC2025 and further enhanced its synergistic effects with 5-FU.

In this study, we demonstrated that targeting ANXA8 reduces ECM deposition by modulating the TCA cycle in DGC. Mechanistically, ANXA8 interacts with SP1 to promote the transcription of PPA1, thereby inhibiting the TCA cycle, which leads to CAF activation and matrix deposition. Deletion of ANXA8 suppresses malignant phenotypes and exhibits synergistic effects with the classical chemotherapeutic agent 5-FU. In conclusion, our findings provide robust preclinical evidence for a promising strategy to improve long-term survival and overcome chemoresistance in patients with DGC.

## Materials and Methods

### Human samples

Human tumor tissue samples were collected from patients at the Second Hospital of Lanzhou University, with written informed consent obtained from each participant. A total of 90 paired diffuse-type gastric cancerous and adjacent normal tissues, collected from patients undergoing radical resection, were included. Among them, 64 patients received 5-FU-based adjuvant chemotherapy after radical resection. This study was approved by the Medical Ethics Committee of Lanzhou University Second Hospital (2024A-297), and written informed consent was obtained from all participants. All procedures followed were in accordance with the ethical standards approved by Institutional Ethics Committee on human experimentation and with the Helsinki Declaration.

### Animal experiments

All mice were raised in the SPF Animal Experimental Center of Lanzhou University Second Hospital under constant humidity (40% to 60%) and temperature (22 to 25 °C). For the PDX model, fresh tumor tissues from a patient with DGC were cut into small pieces (approximately 10 mm^3^) and implanted subcutaneously into 6-week-old female NCG [NOD/Shotput Prkdc^em26Cd52^Il2rg^em26Cd22^/Got mice (GemPharmatech, China, #T001475)]. Following serial transplantation, mice were used for experiments once the average tumor size reached 90 to 100 mm^3^. Samples from patients with DGC were cut into small pieces (diameter 3 mm) and implanted into the axilla of mice. When tumor volumes reached 90 to 100 mm^3^, the mice were randomly divided into several groups, treatment was initiated with 5-FU (MCE, #HY-90006) at 20 mg/kg administered intraperitoneally 2 to 3 times per week, and with UNC2025 (MCE, #HY-12344) or TA@UNC2025 at 75 mg/kg administered orally 2 to 3 times per week. Tumor sizes were measured with calipers 3 times per week, and the experiment was concluded when the tumor volume reached approximately 1,200 mm^3^. Animal study was approved by the Medical Ethics Committee of Lanzhou University Second Hospital (D2024-326). All animal experiments comply with ARRIVE guidelines.

### Human DGC organoids (PDOs)

Human DGC organoids (PDOs) were generated using fresh tumor tissues from a patient with DGC. The tissue was obtained and transported in pre-cooled Human Washing Medium. Upon arrival, the tissue was washed with PBS containing antibiotics, then enzymatically digested with 1 mg/ml collagenase XI (1 mg/ml). The digested material was filtered through a cell strainer, and the digestion was terminated by diluting with Human Washing Medium in a 1:6 ratio. The suspension was then centrifuged at 250 *g* for 5 min, and the supernatant was discarded. The resulting pellet was mixed in a 1:1 ratio with Human Washing Medium and Cultrex UltiMatrix RGF BME (Univ, #BME001-10). The pellet was then resuspended in this mixture. A 50-μl aliquot of the mixture was added to preheated 24-well plates, allowing the BME to solidify. Following solidification, GC organoid medium was added to the wells to support organoid growth.

### Cell culture

The normal human cell line GES-1 and the human GC cell line MKN45 were obtained from the Institute of Basic Medical Sciences, Chinese Academy of Medical Sciences (Beijing, P.R. China). Both cell lines were tested for Mycoplasma contamination and validated by short tandem repeat DNA fingerprinting. GES-1 cells were cultured in Dulbecco's Modified Eagle Medium (Gibco, #C11995500BT) supplemented with 10% fetal bovine serum (FBS) (Gibco, #C0235), while MKN45 and SNU-1 cells were maintained in RPMI-1640 (Gibco, #C11875500BT) with 10% FBS. CAF cell lines were established at our department. The CAF was established from a patient with DGC. The primary culture was initiated as follows: the primary tumor was excised under aseptic conditions and minced with forceps and scissors. The tumor pieces were cultivated in RPMI-1640 (Gibco, #C11875500BT) with 10% FBS (Gibco, #C0235). Serial passages were then carried out every 4 to 7 days. The CAFs used in the experiments were between the 3rd and 12th passage in culture. All cultures included 1% penicillin/streptomycin and were incubated at 37 °C in a 5% CO_2_ atmosphere.

### MST assay

The Monolith NT.115 system (NanoTemper Technologies GmbH, Germany) was utilized to quantify the interaction of ANXA8 (MCE, #HY-P75505) and UNC2025 (MCE), Solithromycin (TargetMol, #T2331), Daclatasvir (TargetMol, #T6229), and Narirutin (TargetMol, #T4S2164). ANXA8 was fluorescently labeled with Kit RED-tris-NTA 2nd Generation (NanoTemper Technologies, #MO-L018). Different concentrations of UNC2025, Solithromycin, Daclatasvir, and Narirutin were co-incubated respectively with ANXA8, followed by MST analysis. The obtained values were then normalized and plotted. Dissociation constants were subsequently determined using a one-point model to fit the curve. The details of the MST assay have been described in this study [58].

### Membrane proteomics

Tumor and adjacent normal tissues obtained from patients with DGC (*n* = 15) were randomly divided into 6 groups, with each group containing either tumor or adjacent normal tissues pooled from 5 patients. Membrane proteins were extracted and purified from the 6 groups of tissues and then analyzed via mass spectrometry-based label-free quantitative proteomics. Human tissue samples were homogenized with SDT lysis buffer, sonicated, and boiled. After centrifugation, the supernatant was filtered, and protein concentration was determined by bicinchoninic acid. SDS-PAGE (sodium dodecyl sulfate-polyacrylamide gel electrophoresis) was used to separate proteins, followed by Coomassie staining. For FASP (filter-aided sample preparation) digestion, proteins were reduced with dithiothreitol, alkylated with iodoacetamide, and digested with trypsin. Peptides were desalted, lyophilized, and quantified by OD280. Mass spectrometry analysis was performed using Easy nLC for chromatographic separation, and the Q-Exactive Plus mass spectrometer for peptide identification. Data were processed using MaxQuant for database searching and quantification. Calibration curves were constructed for quantification, with lower limit of detection (LLOD) and lower limit of quantitation (LLOQ) determined by signal-to-noise ratios. Accuracy and precision were validated using recovery percentages and relative standard deviations (RSDs) from QC samples. Quantification was based on the final concentration, considering dilution factors and sample volume. A total of 32,187 peptides and 4,293 proteins were identified. The data from at least 2 non-missing values in the 3 replicate experiments within each sample group were subjected to statistical analysis. Proteins that met the criteria of *P* < 0.05 and |log_2_FC| > 1 were considered differentially expressed proteins. GSEA was performed to evaluate the significance of protein enrichment in a specific GO term or KEGG pathway in tumor tissues compared to adjacent normal tissues.

### Central carbon metabolite analysis

This analysis was conducted by BIOTREE (Shanghai, China). Human GC cell samples were collected by the cell scraping method: aspirate the cell culture fluid, remove the culture medium, and wash it quickly with pre-cooled PBS solution for 2 to 3 times; scrape the cells into a centrifuge tube with a cell scraper; place the centrifuge tube containing the sample into liquid nitrogen for quenching for 1 min; remove the sample and transfer it to a refrigerator at −80 °C or liquid nitrogen for freezing. The samples were extracted with MeOH/H_2_O, followed by freeze–thaw and sonication, and centrifuged to collect the supernatant for analysis. The HPIC-MS/MS system with a Dionex ICS-6000 was used for separation, and an AB SCIEX 6500 QTRAP+ mass spectrometer with MRM was employed for quantification. Calibration curves were fitted using least squares regression, with LLODs and LLOQs determined based on signal-to-noise ratios. Analytical recoveries ranged from 91.6% to 116.9%, with RSDs below 14%. The method demonstrated high accuracy and precision for metabolite quantification in biological samples. The filtering of individual metabolites was performed by retaining only those with no more than 50% missing values in a single group or no more than 50% missing values across all groups. Missing values in the original data were imputed using a simulation method (missing value recoding), where the missing values were filled by multiplying the minimum value by a random number between 0.1 and 0.5. After preprocessing, 47 metabolites were retained, and those that met the criteria of *P* < 1 and VIP > 0 (VIP: variable importance in projection from the OPLS-DA model) were considered as differentially expressed metabolites. The KEGG Pathway database (http://www.kegg.jp/kegg/pathway.html) was used to map the differential metabolites to all pathways corresponding to the species *Homo sapiens* (human).

### Statistical analysis

Statistical analyses were performed using SPSS 27.0, R 4.3.3, and GraphPad Prism 9.5. Shapiro–Wilk test was used to assess the normality of the data. For normally distributed samples, Student’s *t* test, one-way analysis of variance (ANOVA), and Welch’s ANOVA were used to compare differences between 2 and multiple groups, respectively. Subsequently, post hoc analysis was conducted using Tukey’s multiple comparisons test or Tamhane’s T2 multiple comparisons test, respectively. For some of the repeated measurements that did not follow a normal distribution and were complex, we employed generalized estimating equations for statistical analysis. Non-normally distributed values were assessed using nonparametric tests. Survival analysis was conducted using Kaplan–Meier curves, and the log-rank test was used to assess survival outcomes.

Multivariate Cox models were employed to assess the impact of assumed risk factors on the OS of patients with high ANXA8. All tests were 2-sided, and statistical significance was set at *P* < 0.05. In vitro assays were repeated at least thrice with biological and technical replicates, and *n* represents the number of independent biological replicates per group.

A detailed methodological study is available in the Supplementary Materials.

## Data Availability

All other data are available in the article and the Supplementary Materials or from the corresponding authors upon reasonable request.
