# Erratum to “Targeting the ANXA8–SP1–PPA1 Axis to Modulate TCA Cycle and Matrix Deposition in Diffuse-Type Gastric Cancer”

**DOI:** 10.34133/research.1395

**Published:** 2026-08-05

**Authors:** Yuxia Wu, Xiangyan Jiang, Huiguo Qing, Yansong Hou, Yong Ma, Tao Wang, Keshen Wang, Long Qin, Weiwen Cai, Zongrui Xing, Bin Zhao, Qichen He, Wenbo Liu, Tian Wang, Haonan Sun, Xingshuo Zhao, Zuoyi Jiao, Zeyuan Yu

**Affiliations:** ^1^ Department of General Surgery, Lanzhou University Second Hospital, Lanzhou, Gansu, China.; ^2^ TheSecond School of Clinical Medicine, Lanzhou University, Lanzhou 730000, China.; ^3^ Cuiying Biomedical Research Center, Lanzhou University Second Hospital, Lanzhou 730030, China.; ^4^ Gansu Province High-Altitude High-Incidence Cancer Biobank, Lanzhou University Second Hospital, Lanzhou 730030, China.; ^5^ Gansu Tumor Immunology Basic Disciplines Research Center, The Second Hospital & Clinical MedicalSchool, Lanzhou University, Gansu 730030, China.

In the Research Article “Targeting the ANXA8–SP1–PPA1 Axis to Modulate TCA Cycle and Matrix Deposition in Diffuse-Type Gastric Cancer,” the authors have identified errors in Figures 3 and 4 [1].

The representative image for SDHA, which should have appeared in Figure 4, was mistakenly used in Figure 3H. The authors have replaced the incorrect image in Figure 3H with the correct representative image of Low ANXA8. Additionally, a similar error was identified in the representative H&E staining image of cardiac tissue from the 5-FU group. After rechecking the original data, we replaced it with the correct representative image. The corrected figures are below and have also been updated in the original HTML and PDF. The authors deeply apologize for these errors and want to reassure readers that all corrections are based on validated original data. Importantly, these errors do not affect the integrity of the research data, analysis, or the main conclusions.

**Fig. 3.**
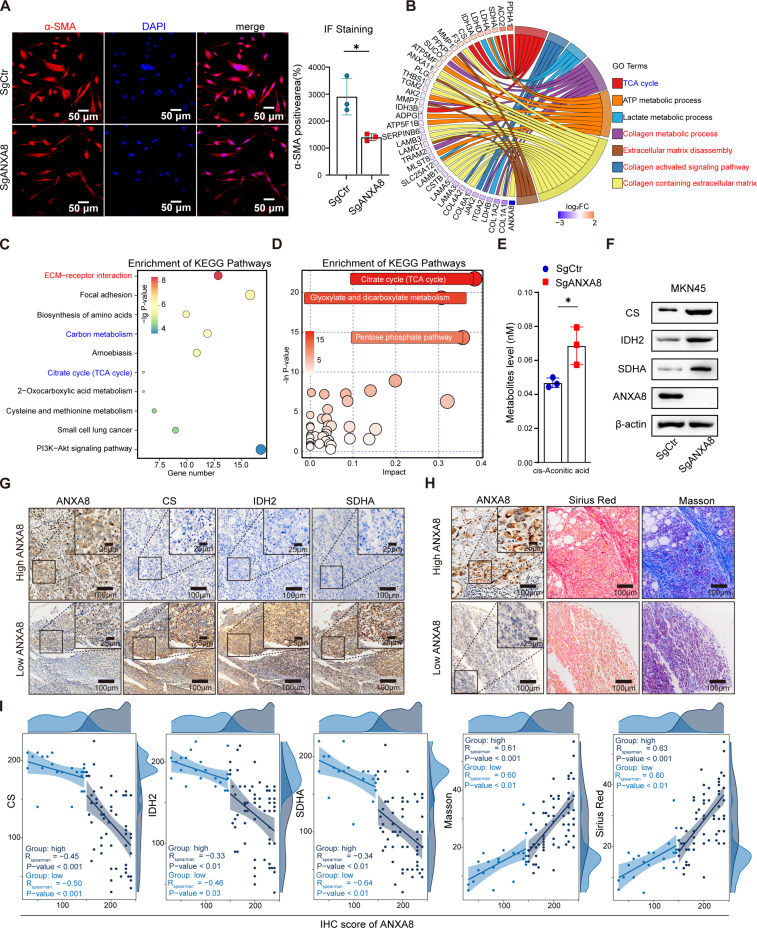
ANXA8 inhibits TCA cycle and aberrant ECM deposition in DGC. (A) Representative fluorescence microscopy images and quantitative statistics illustrating the effect of SgANXA8 on CAFs (original magnification ×20; n = 3). Scale bar, 50 μm. (B) GO functional enrichment analysis of differentially expressed proteins between SgANXA8 and control MKN45 cells. (C and D) KEGG pathway enrichment analysis of differentially expressed proteins and metabolites between SgANXA8 and control MKN45 cells. (E) Quantitative assessment of cis-aconitic acid levels. (F) Quantitative statistics of immunoblotting assay showing protein levels of CS, IDH2, SDHA, ANXA8, and β-actin in MKN45 cells. (G to I) Representative images and correlation analysis of IHC staining for CS, IDH2, SDHA, and Masson’s and Sirius Red staining in patients with DGC exhibiting high (IHC score ≥150, n = 67) versus low (IHC score <150, n = 23) ANXA8 expression. Scale bar, 100 and 25 μm. Quantitative statistics showed a Spearman correlation between CS, IDH2, SDHA, Masson’s, Sirius Red, and ANXA8; a fitted line represents the linear regression model. Results are presented as mean ± standard deviation in (A), (E), and (F). n: Biological duplication per group. Student’s t test, *P < 0.05. GO, Gene Ontology.

**Fig. 4.**
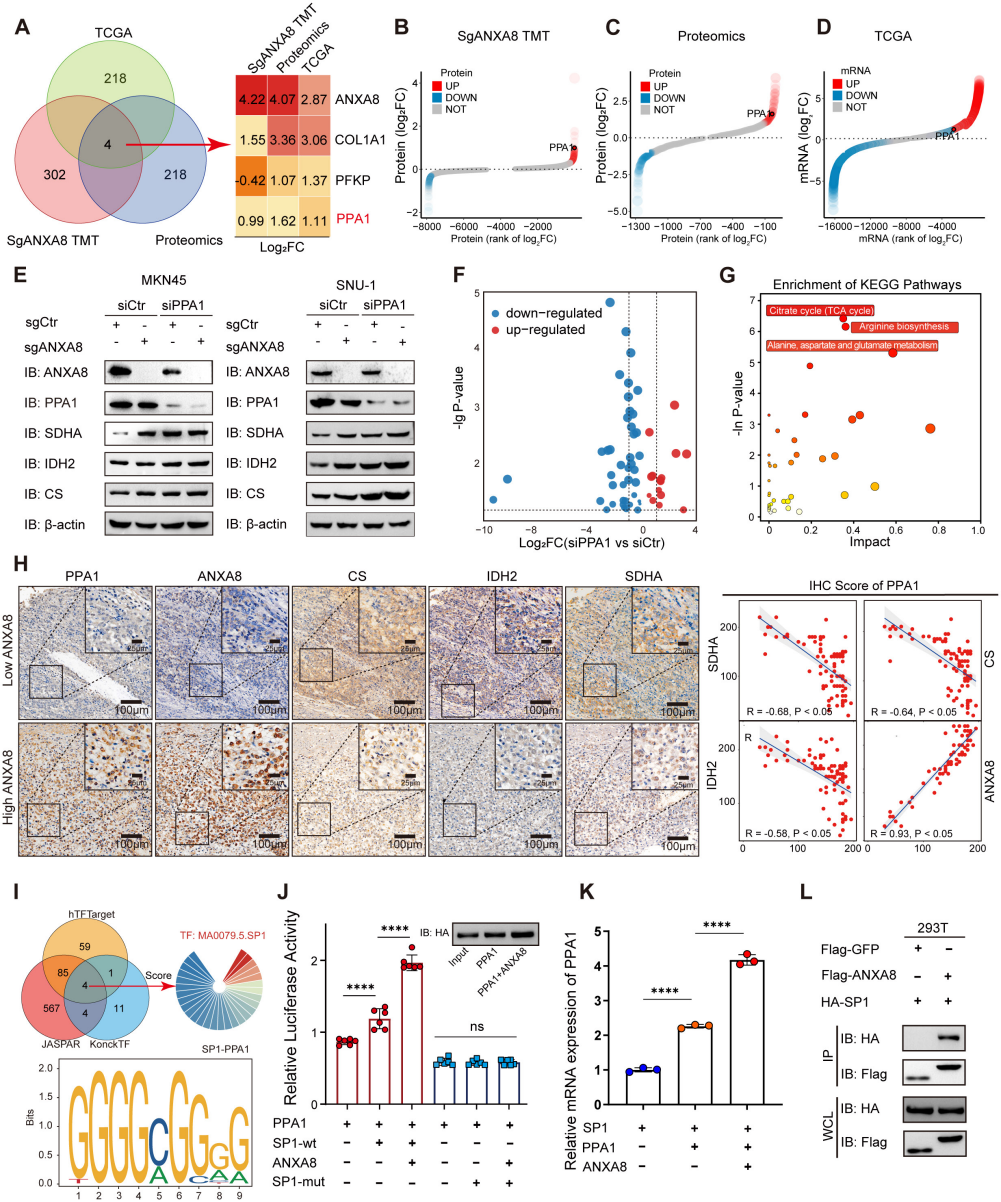
ANXA8 regulates the TCA cycle and ECM through interaction with PPA1 and SP1. (A) Venn diagrams and heatmaps show the overlap between proteomics, SgANXA8TMT data, and TCGA mRNA data. (B) TMT data comparing SgANXA8 with SgCtr in MKN45 cells. (C) TCGA mRNA data comparing DGC with NATs. (D) TCGA data comparing DGC with NATs. (E) Immunoblotting analysis of CS, IDH2, SDHA, ANXA8, PPA1, and β-actin in SgCtr and SgANXA8 cells, with or without siPPA1 in MKN45 and SNU-1 cells. (F and G) Differentially expressed metabolites and KEGG functional enrichment analysis. (H) Representative images and correlation analysis of IHC staining for ANXA8, CS, IDH2, and SDHA in DGC patients with high (IHC score ≥150, n = 58) versus low (IHC score <150, n = 32) PPA1 expression. Scale bar, 100 and 25 μm. Quantitative statistics reveal a Spearman correlation between ANXA8, CS, IDH2, SDHA, and PPA1, with a fitted line representing the linear regression model. (I) Venn diagrams and heatmaps reveal the overlap TF of PPA1 between hTFTarget, JASPAR, and KonckTF data. The most marked score binding with the PPA1 promoter is MA0079.5.SP1, PPA1 motif (bottom).(J) DNA pull down assay showing the interaction of SP1 with PPA1 promoter (top), and dual-luciferase gene reporter assays detecting the transcriptional activity of the indicated PPA1 promoter with or without SP1 and ANXA8 overexpression with or without SP1 mut (bottom). (K) Quantitative real-time PCR (QRT-PCR) assays were employed to detect the effects of ANXA8, and SP1 on PPA1 expression. (L) Co-immunoprecipitation (Co-IP) assays were employed to assess the interaction of ANXA8 with SP1 in 293Tcells expressing the indicated plasmids, with GFP serving as a control. Results are presented as mean ± standard deviation in (E), (J), and (K). n: Biological duplication per group. Student’s t test, ****P < 0.0001, ns: not significant.
