# Supplementary material for: Targeting the ANXA8–SP1–PPA1 Axis to Modulate TCA Cycle and Matrix Deposition in Diffuse-Type Gastric Cancer
Source: Research (Wash D C). 2025 Aug 25;8:0838. doi: 10.34133/research.0838 (PMC12377485; doi:10.34133/research.0838)
Supplement: Supplementary 1 — Materials and Methods Figs. S1 to S16 [file research.0838.f1.zip › supplementary materials and methods.pdf]

Cell Viability Assay

Approximately 4000 cells per well were seeded in 96-well plates and incubated overnight to allow for cell attachment. The cells were then treated with varying concentrations of 5-FU and UNC2025 for 72 h. Cell viability was assessed by measuring the optical density at 450 nm (OD450) after a 2h incubation with Cell Counting Kit-8 (CCK-8) (Selleck, #B34302). For viability detection in DGC organoids, approximately 100 spheroids per well were suspended in a mixture of BME and medium, then seeded in 96-well plates. The viability of organoids was determined after 7 days of treatment with 5-FU and UNC2025 using the CellTiter-Glo® 3D Cell Viability Assay Kit (Promega, #G9683). The IC50 values were calculated using GraphPad Prism 9.0 software.

Colony Forming Assay

Approximately 2000 cells were seeded in 35 mm-diameter dishes and allowed to grow for 72 h. The cells were then treated with 5-FU and UNC2025 at the specified concentrations for an additional 72 h. Colonies were subsequently fixed in 4% paraformaldehyde and stained with 0.05% crystal violet for visualization. For the organoid growth assay, organoids were cultured for 3 days, after which images were captured using phase contrast microscopy with an OLYMPUS IX53 microscope (Olympus Corporation, Japan).

Transwell invasion assay

MKN45 cells (3×10^5) were seeded into the upper chamber of a Matrigel-coated transwell plate (Corning 3422; Matrigel: BD Biosciences, 354248). Serum-free medium was added to the upper chamber, while medium containing 10% FBS was placed in the lower chamber as a chemoattractant. After 48 h of incubation, non-invading cells were gently removed from the upper surface of the Matrigel with a cotton swab. The cells that had invaded through the Matrigel were fixed with 4% paraformaldehyde, stained with crystal violet (Solarbio, C8470), and imaged using an Olympus IX53 microscope. The data were analyzed using ImageJ software (v1.49).

Drugs synergy evaluation

Based on the IC50 values for human DGC organoids and the solubility profiles of 5-FU and UNC2025, six concentration gradients were established for UNC2025 and 5-FU. Organoids were seeded in 96-well plates, and the corresponding drugs were added according to the different concentration combinations once the organoids formed a distinct ring structure. After 6 days of continuous treatment, organoid viability was assessed using the CellTiter-Glo® 3D Cell Viability Assay (Promega, #G9682) following the manufacturer's instructions. The synergistic therapeutic effects of 5-FU and UNC2025 on human DGC organoids were evaluated using Combenefit software and the Synergy Finder web application (version 3.0). The three-dimensional visualization of the synergistic effect was generated using the LOEWE, HSA, and BLISS models in Combenefit software. Synergy scores based on the LOEWE, ZIP, HSA, and BLISS models were calculated using the Synergy Finder web application.

Immunohistochemistry (IHC)

IHC staining was performed to detect the expression of CS, IDH2, SDHA, PPA1 and ANXA8 in DGC tissues. TMA were deparaffinized, rehydrated, and subjected

to antigen retrieval. The slides were then incubated with primary antibodies: anti-CS (Abcam, 1:100, #ab129095, RRID: AB_11143209), anti-IDH2 (Abcam, 1:100, #ab131263, RRID: AB_11156098), anti-SDHA (Cell Signaling Technology, 1:100, #11998, RRID: AB_2750900), anti-PPA1 (Proteintech, 1:100, # 14985-1-AP, RRID: AB_2167890), and anti-ANXA8 (Abcam, 1:100, #ab111708, RRID: AB_10861107). Following incubation, the slides were dehydrated, mounted with a stabilizing medium, and imaged using a KF-PRO-120 scanner (Konfoong). The staining intensity (scored as 0, 1, 2, or 3) and the percentage of positive cells (ranging from 0% to 100%) were independently evaluated by two pathologists. The final H-score was calculated by multiplying the staining intensity by the percentage of positive cells.

Hematoxylin and Eosin Staining

Slides of organoids and TMA were deparaffinized by three washes in xylene, followed by rehydration through graded ethanol. The slides were then stained with hematoxylin, rinsed in water and ethanol, and counterstained with 1% eosin. After staining, the slides were dehydrated using graded ethanol and xylene washes, and then sealed with coverslips.

Immunofluorescence and Confocal Microscopy Analysis

Cells cultured on 35 mm glass-bottom dishes (MatTek Corporation) were fixed with 4% paraformaldehyde for 10 minutes and permeabilized with 0.1% Triton X-100. Following permeabilization, cells were blocked with 3% BSA for 1 h at room temperature and then incubated overnight at 4°C with a primary antibody against α-SMA (Sigma-Aldrich, 1:100, A2547, RRID: AB_476701). After three PBS washes, the cells were incubated with the appropriate fluorescent-labeled secondary antibody and counterstained with DAPI. Imaging was performed using a Zeiss LSM 880 laser microscope (×25 objective; Plan-Apochrom 1.4). Quantification of α-SMA positivity was conducted using ImageJ software (version 1.53c).

Immunoblotting

Cells or tissues were lysed in RIPA buffer and centrifuged at 12,000 × g for 10 minutes. The extracted proteins were separated by SDS-PAGE and transferred onto membranes. Membranes were incubated overnight at 4°C with primary antibodies: anti-CS (Abcam, 1:1000, #ab129095), anti-IDH2 (Abcam, 1:1000, #ab131263), anti-SDHA (Cell Signaling Technology, 1:1000, #11998), anti-ANXA8 (Abcam, 1:1000, #ab111708), anti-FLAG (Sigma,1:1000, #F1804), anti-HA (Invitrogen,1:1000, #71-5500) or β-actin (Abcam, 1:1000, #ab8226). Secondary antibodies conjugated to horseradish peroxidase were then applied, and detection was performed using enhanced chemiluminescence with a MiniChemi 610 Plus imaging system (Beijing Saizhi Venture Technology Co., Ltd.). The ratio of secondary antibody of goat anti-mouse IgG (Bioworld, #BS12478) or anti-rabbit IgG (Bioworld, #BS13278) was 1:10000.

Collagen Staining and Quantification

Tumor sections were deparaffinized and dehydrated before being stained with Sirius Red and Masson’s Trichrome. After staining, sections were counterstained with hematoxylin, followed by dehydration and sealing. Stained tumor sections were scanned using the TISSUE FAXS PLUS Panoramic Tissue Quantification System, and the collagen fiber area was quantified using ImageJ software.

Quantitative real-time PCR

Total RNA was extracted from harvested cells using TRIzol reagent (Invitrogen) and reverse transcribed into cDNA using a reverse transcription kit (Takara, #RR037A), following the manufacturer's instructions. QRT-PCR was performed using a Rotor-Gene Q instrument (QIAGEN) with TB Green dye (Takara, #RR420Q) and 10 μmol/L of forward and reverse primers. GAPDH (Forward primer: CTGGGCTACACTGAGCACC, Reverse primer: AAGTGGTCGTTGAGGGCAATG) was used as a control to analyze the transcriptional expression of CS (Forward primer: TGCTTCCTCCACGAATTTGAAA, Reverse primer: TGCTTCCTCCACGAATTTGAAA), IDH2 (Forward primer: CGCCACTATGCCGACAAAAG, Reverse primer: ACTGCCAGATAATACGGGTCA), SDHA (Forward primer: TGGCATTTCTACGACACCGTG, Reverse primer: GCCTGCTCCGTCATGTAGTG), and SP1 (Forward primer: GTGGAGGCAACATCATTGCTG, Reverse primer: GCCACTGGTACATTGGTCACAT).

Plasmids and small interfering RNAs

pCMV3-FLAG-ANXA8 (HG15421-CF) and pCMV3-HA-SP1 (HG120024-CY) were purchased from Sino Biological Inc. (Beijing, China). The cDNA sequences of a promoter region of PPA1 were then cloned into the dual luciferase vector pGL4.10-hRluc (hedgehogBio, #HH-LUC-043, Shanghai, China) for dual luciferase reporter gene assay. GenOFF small interfering RNAs for ANXA8 (#siB07122116540-1-5), PPA1 (#siG000005464A-1-5) and corresponding controls (#siT0000001-1-5) were procured from Guangzhou 48 RiboBio (Guangzhou, China) and transfection was conducted in accordance with the manufacturer's instructions.

Dual-luciferase reporter gene assay

The most score binding motif of SP1 and PPA1 promoter was predicted by hTFTarget, JASPAR and KonckTF database. The full length, most score binding motif and PPA1 promoter sequences were cloned into the dual luciferase vector pGL-4.2.2. HEK-293T cells were seeded in 24-well plates and 200 ng of the dual-luciferase reporter plasmids with the full length and most score binding motif of the PPA1 promoter, pCMV3-FLAG-ANXA8 and pCMV3-HA-SP1 were co-transfected using Lipofectamine 2000 when the cells were 70% confluent. After 24 hours, firefly luciferase activity and Renilla luciferase activity were detected using the Dual-Luciferase Reporter Assay System (Promega, #E1910) according to the manufacturer's instructions.

DNA pull down assay

The DNA pull down kit (BersinBio, #Bes5004, Guangzhou, Beijing) was used to find the transcription factors that bind to the PPA1 promoter domain. Firstly, DNA probes for the PPA1 promoter region were designed and labelled with desulphurised biotin by Sangon Biotech (Shanghai, China). Subsequently, the streptavidin-coupled magnetic beads were specifically bound to the desulfobiotin-labelled DNA probe to produce a DNA probe-magnetic bead complex. The nuclear protein of MKN45 cells was extracted, nucleic acid was removed, and the DNA probe-magnetic bead complex was incubated with the extracted nuclear protein. The potential transcription factor PPA1 was able to bind specifically to the DNA probe. After

washing, non-specific binding protein molecules were removed. Finally, the DNA probe-protein complex was eluted with streptavidin, and the PPA1 transcription factor was identified by immunoblotting or mass spectrometry.

Bioinformatics analysis of gene expression profiles in DGC

The mRNA and clinical data of DGC were downloaded from TCGA database (https://cancergenome.nih.gov) and GEO database (https://www.ncbi.nlm.nih.gov/geo/). Data analysis and visualization can be optimized with the Limma, clusterProfiler, MsigDB and ggplot2 packages in R-4.3.3.

scRNA-seq processing, quality control, dimensionality reduction, clustering, visualization and differential gene expression analysis

Single-cell RNA sequencing (scRNA-seq) data were analyzed using two independent datasets (GSE183904 and GSE150290) with the Seurat R package (version 5.0.1). The combined dataset consists of 377,343 raw single cells, with 261,807 high-confidence cells retained after quality control (QC). The data were categorized into the following groups: DGC (n = 79,638 cells), DGC NAT (n = 53,502 cells), IGC (n = 79,503 cells), IGC NAT (n = 26,377 cells), and Normal (n = 22,787cells). Quality control thresholds were set as follows: nUMI > 500, nGene > 250, log10GenesPerUMI > 0.8, mitoRatio < 0.1, and genes expressed in at least 10 cells were retained. Batch-effect correction was applied using the Harmony algorithm to ensure data consistency across the integrated datasets. The data matrix was normalized for sequencing depth by dividing the total number of UMIs for each cell, followed by log-transformation using the Seurat package. Highly variable genes were identified, and batch effects were corrected using the ScaleData function. Principal component analysis (PCA) was performed with the top 20 principal components, and clustering was carried out at a resolution of 0.8 using the FindClusters function. Cell types were annotated with SingleR and cell marker. Differentially expressed genes (DEGs) were determined using the FindAllMarkers function with the Wilcoxon rank-sum test. Genes expressed in at least 10% of cells and with $\log_2$ fold change ($\log_2$FC) ≥ 0.25 were considered. For KEGG enrichment analysis, cancer epithelial cells were compared with paired NAT-derived epithelial cells as internal controls to minimize inter-individual variation. In the ECM analysis, the focus was on fibroblasts. Malignant ECM markers (ACTA2, FAP, MMP11, INHBA, POSTN, TNC, SERPINE1, LOX) were found to be elevated in normal adjacent tissues, and normal tissues were used as controls for this analysis.

Proteome analysis

This proteome analysis was conducted by BIOTREE (Shanghai, China). Nano-UPLC (EASYnLC1200) was connected to a Q Exactive HF-X (ThermoFisher) for LC-MS analysis. Peptides were separated using a reversed-phase column and a 90-minute gradient. Data-dependent acquisition (DDA) was performed with an Orbitrap analyzer at 120,000 resolution (MS1) and 45,000 (MS2). Peptide identification utilised SEQUEST HT with UniProt databases, with TMT Pro tags for quantification. Normalisation was based on total peptide amounts, with a false discovery rate set at 1%. All parameters were set to default where not specified.

Lentiviral production and infection

HEK293T cells were used for lentiviral production. Modified plasmids and

lentiviral packaging plasmids (pSPAX2 and pMD2G) were co-transfected into the cells using PEI 40K Transfection Reagent (Servicebio, #G1802). Lentiviruses were harvested 48 h post-transfection and filtered through a 0.45-μm non-pyrogenic filter (Biosharp, #BS-PES-45). MKN-45 cells and organoids were infected with the lentiviruses in the appropriate medium. Transduced cells were selected using 3μg/mL puromycin. The sequences of guide RNAs (SgRNAs) of ANXA8 were as follows: SgRNA-#1 (Forward primer: CCCTCACCAGTGTGTCACCC, Reverse primer: GGGTGACACACTGGTGAGGG), SgRNA-#2 (Forward primer: CTTCAAGGCTCAGTTCGGCA, Reverse primer: TGCCGAACTGAGCCTTGAAG) and SgRNA-#3 (Forward primer: GCCATACAGATACGAAGCCA, Reverse primer: TGGCTTCGTATCTGTATGGC).

Preparation of TA@UNC2025

UNC2025, PEG300-HA, and TA were fully dissolved in DMSO at 25°C to prepare a stock solution. The solution was stirred overnight to allow for self-assembly. Following stirring, the solution was placed in a dialysis bag (MWCO: 1000 Da, Fisher Scientific, #08-700-197) and dialyzed against ultrapure water for 48 hours. The resulting solution was then lyophilized using a freeze dryer for subsequent use. The Tyndall effect was observed in the self-assembled solution, indicating successful formation.

Characterization of TA@unc2025 nanoparticles

The morphology of TA@UNC2025 nanoparticles was examined using transmission electron microscopy (TEM, Hitachi, Japan). A small aliquot of the TA@UNC2025 aqueous solution was applied to a carbon-coated copper grid, and the excess solution was allowed to dry for 5 minutes before TEM analysis. The hydrodynamic diameter of TA@UNC2025 was assessed using dynamic light scattering (DLS) at 25°C with a 90Plus PALS instrument (Brookhaven Instruments Corporation, USA). The stability of TA@UNC2025 particles was also evaluated via DLS.

UV-vis absorbance spectra of PEG300-HA, TA, UNC2025, and TA@UNC2025 in DMSO were recorded using a UV-vis spectrophotometer (SHIMADZU, Japan). The UNC2025 content in TA@UNC2025 was quantified using an F320 Fluorescence Spectrophotometer (ANGDONG, China), with a calibration curve of UNC2025 in DMSO. Fourier transform infrared spectroscopy (FT-IR) was performed on a Thermo Fisher Nicolet iS5 infrared spectrometer (Bruker, Karlsruhe, Germany) over a range of 4000 cm^{-1} to 400 cm^{-1}.

In Vitro UNC2025 Release from TA@UNC2025 Nanoparticles. The in vitro release of UNC2025 from TA@UNC2025 nanoparticles was evaluated in PBS at pH 7.4 and pH 5.0, using a dialysis bag (MWCO: 1000 Da) at 37°C in an incubator shaker. A predetermined volume of TA@UNC2025 nanoparticle solution (10 mL) was placed in the dialysis bag and immersed in 100 mL of the respective buffer solution. At predetermined time intervals, 5 mL of the incubated solution was withdrawn, and an equal volume of fresh buffer was added to maintain a constant volume. The release profiles of UNC2025 were characterized by measuring the fluorescence intensity of the solutions at 285 nm, using a calibration curve of UNC2025 in the same PBS buffer.
