# Supplementary material for: Targeting the ANXA8–SP1–PPA1 Axis to Modulate TCA Cycle and Matrix Deposition in Diffuse-Type Gastric Cancer
Source: Research (Wash D C). 2025 Aug 25;8:0838. doi: 10.34133/research.0838 (PMC12377485; doi:10.34133/research.0838)
Supplement: Supplementary 1 — Materials and Methods Figs. S1 to S16 [file research.0838.f1.zip › supplementary figures S1 to S16--clean version.pdf]

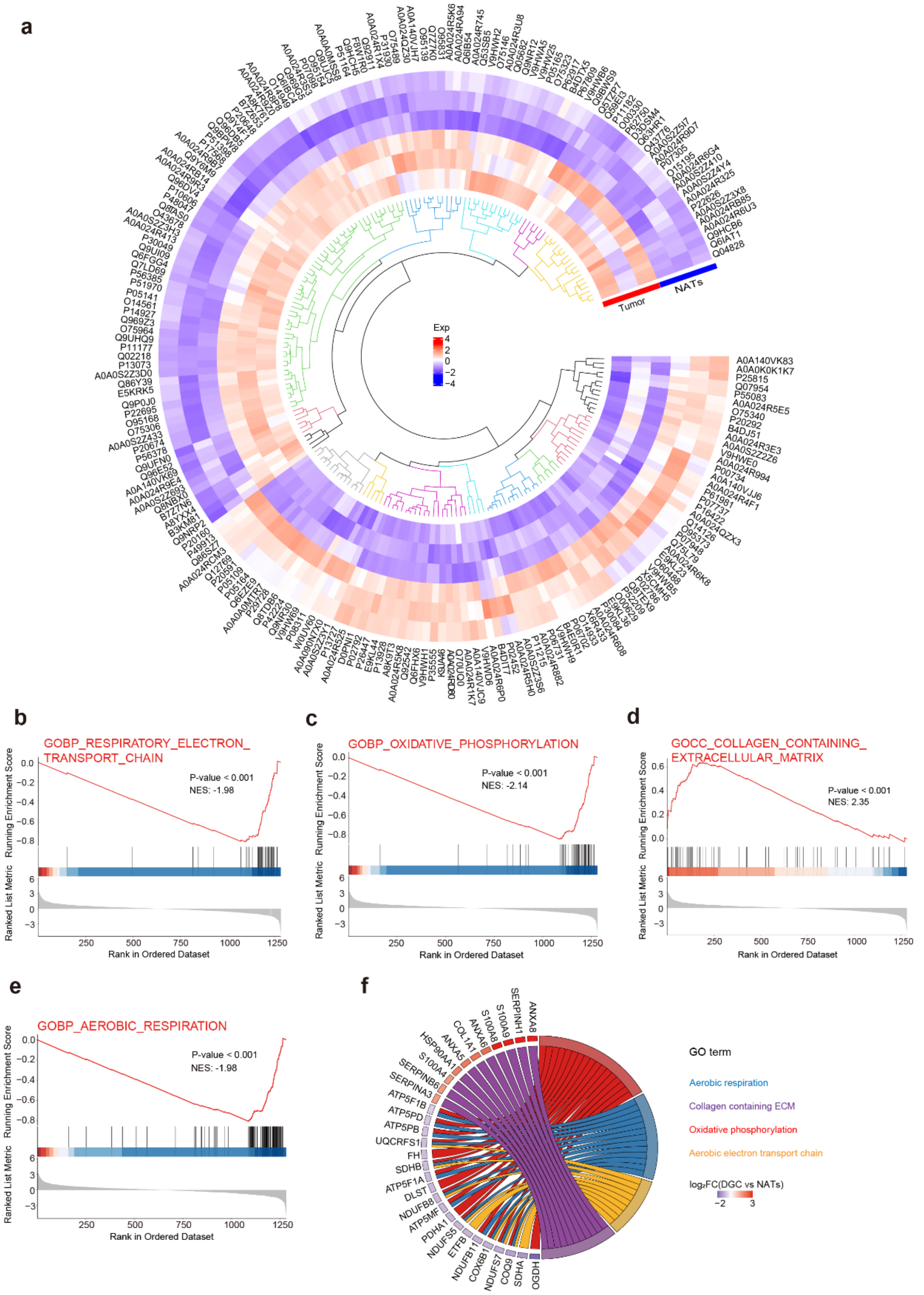

Supplementary Figure 1. Proteomics analysis in Diffuse-type Gastric Cancer

a: Heatmap of differentially expressed proteins between patient-derived tumor and paired normal adjacent tissues (NATs). b-f: GO enrichment maps of aerobic electron transport chain, oxidative phosphorylation, collagen containing ECM, and aerobic respiration pathways based on GSEA analysis, with a 25% false discovery rate (FDR) and P < 0.05.

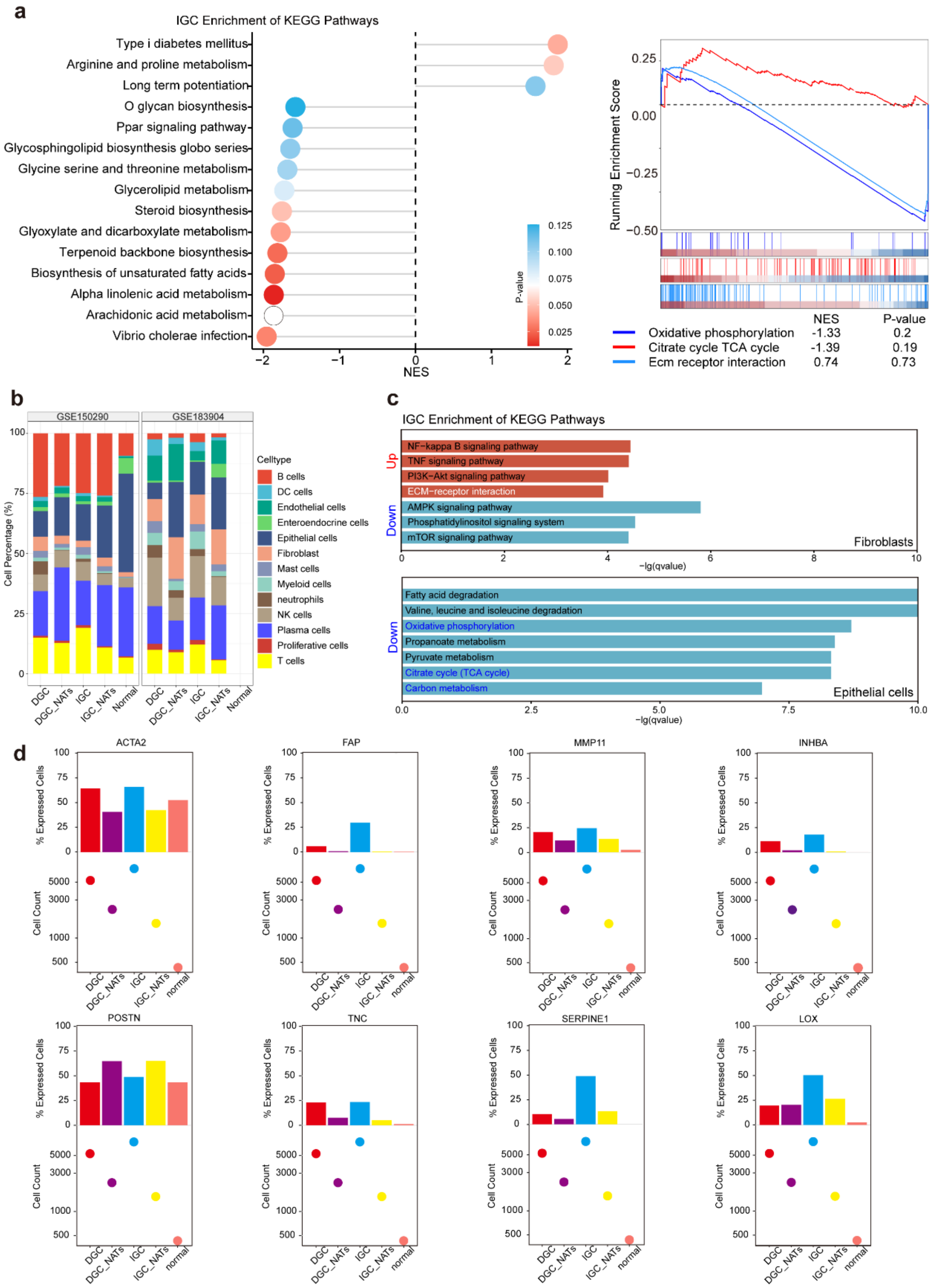

Supplementary Figure 2. Enrichment analysis in Gastric Cancer

a: Pathway changes of IGC in TCGA data. b: Scale plot of cells in scRNA data in GC. c: Pathway changes of IGC in scRNA data. d: Expression of malignant ECM markers among different tissues.

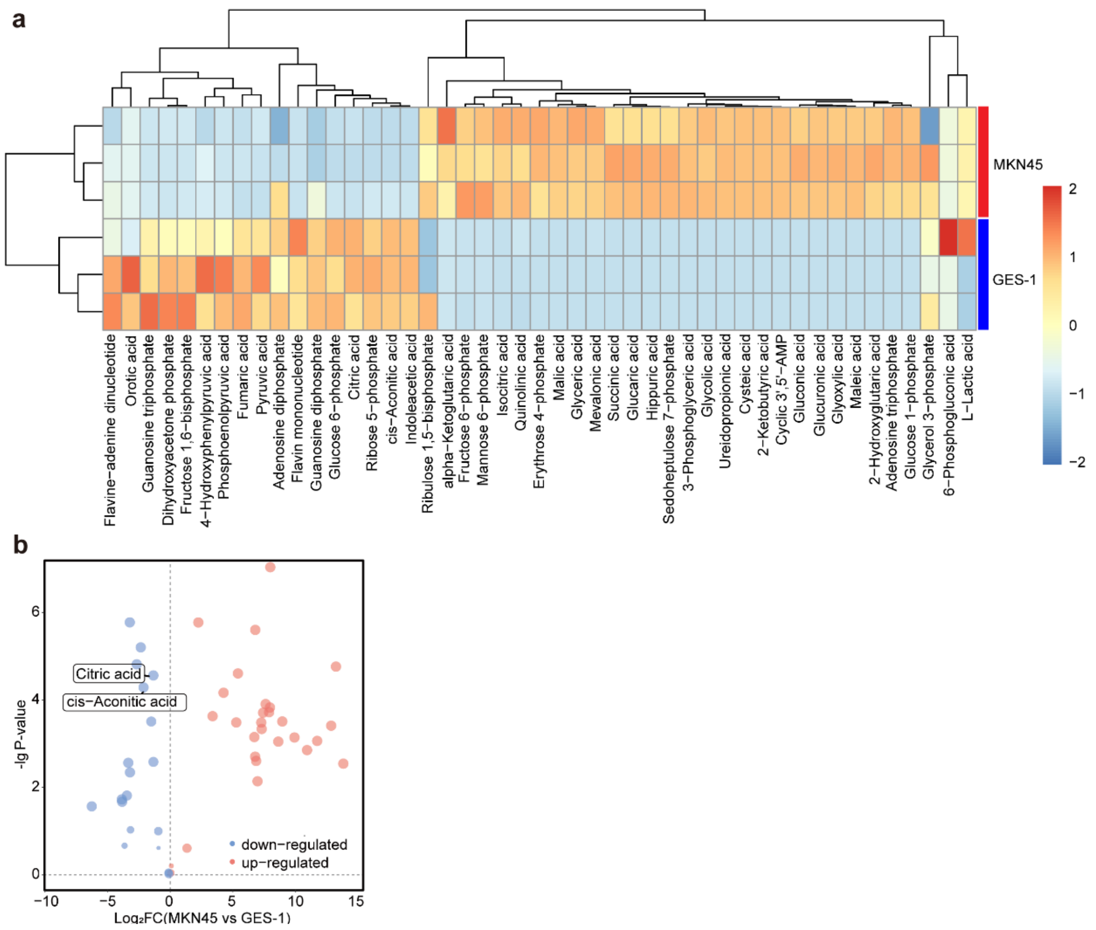

Supplementary Figure 3. Metabolomics were performed in GES-1 cells versus MKN45 cells

a and b: The volcano plot and Heatmap plot showing differentially expressed metabolites between GES-1 cells and MKN45 cells.

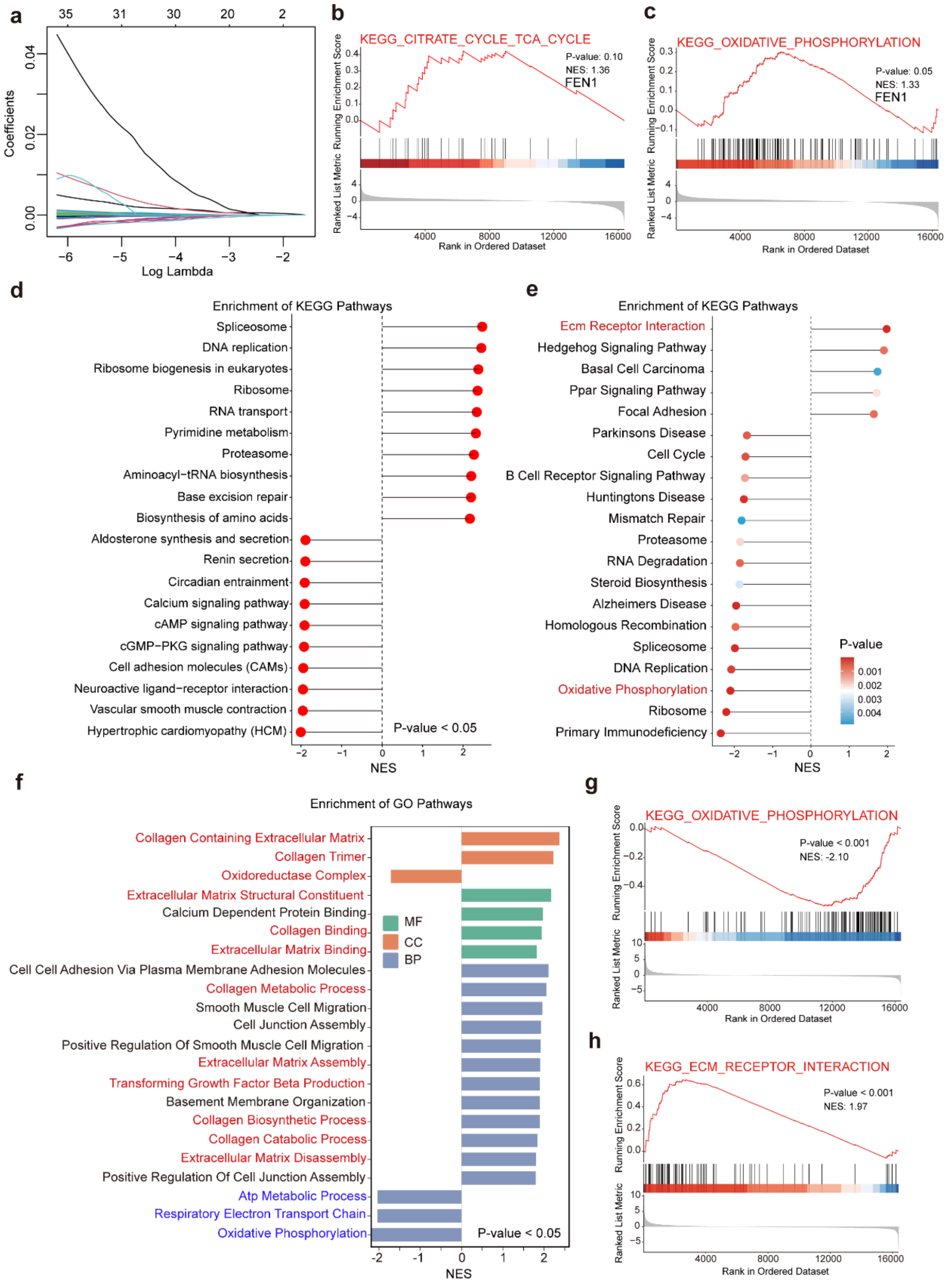

Supplementary Figure 4. Selecting genes associated with the TCA Cycle

a: LASSO coefficient profiles for 83 mRNAs, with the optimal value based on the minimum criterion yielding two non-zero coefficients. b and c: Enrichment maps of the TCA cycle and oxidative phosphorylation pathway based on GSEA analysis for the FEN1, with a 25% false discovery rate (FDR) and P < 0.05. d: KEGG pathway analyses of FEN1-associated genes based on GSEA analysis in LinkedOmics data, with a 25%

FDR and P < 0.05. e-h: KEGG and GO pathway analyses of ANXA8-associated genes based on GSEA analysis, enrichment maps of oxidative phosphoylation and ECM-receptor interaction pathways based on GSEA analysis in TCGA data, with a 25% FDR and P < 0.05.

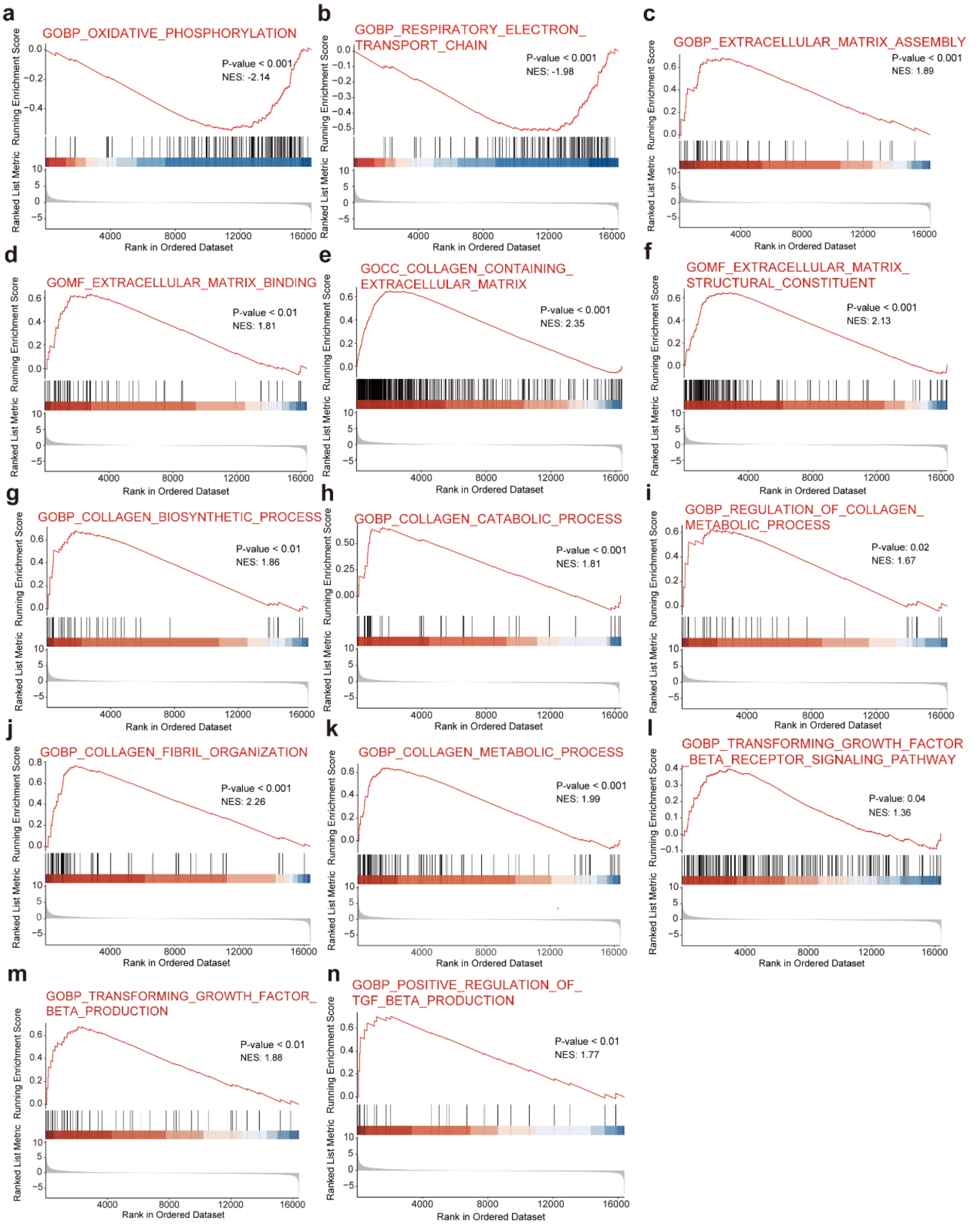

Supplementary Figure 5. Enrichment Analysis of ANXA8

a-n: Enrichment maps of GO pathways based on GSEA analysis in TCGA data, with a 25% FDR and P < 0.05.

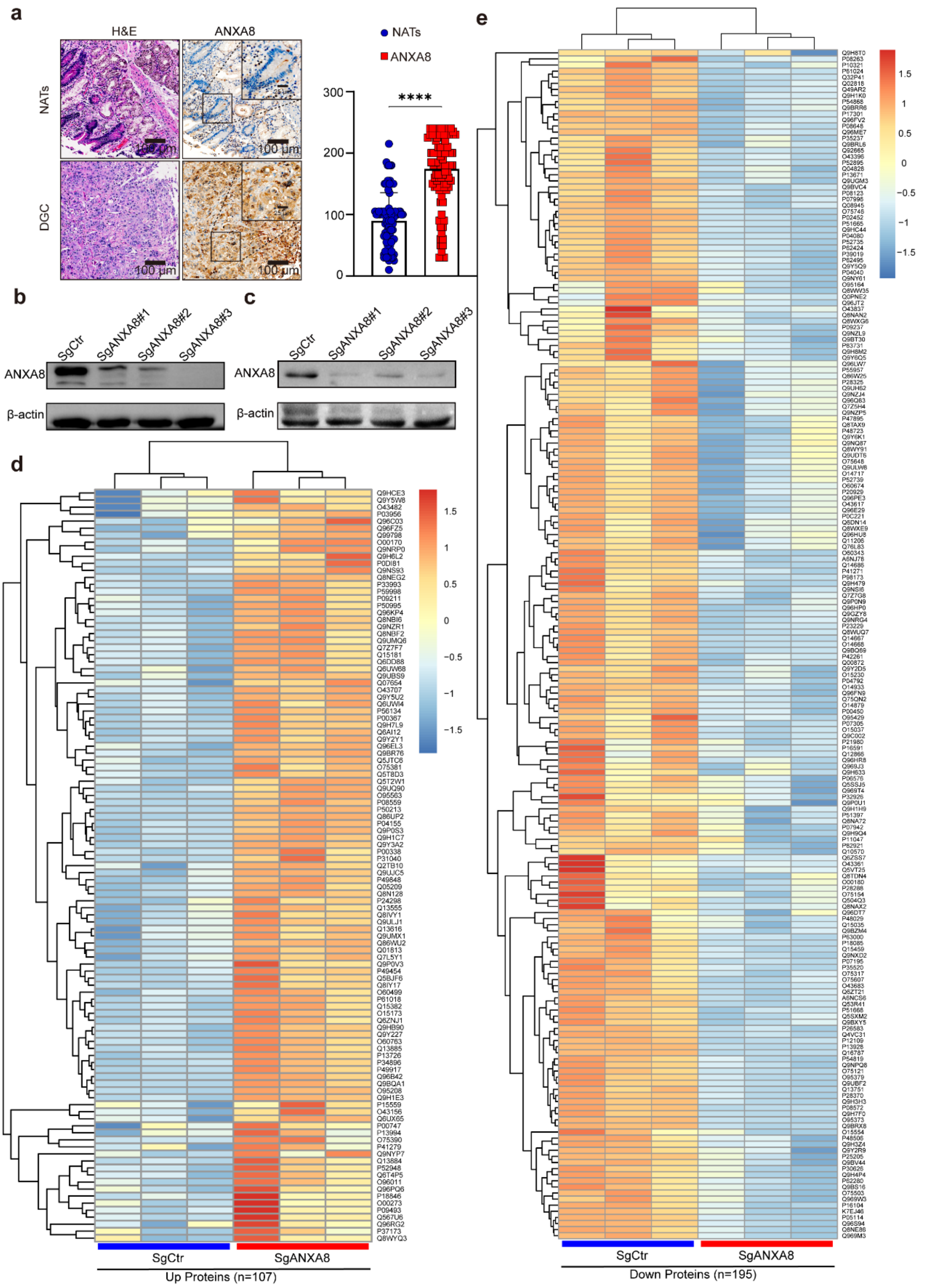

Supplementary Figure 6. Proteomics analysis between control and SgANXA8 MKN45 cells

a: Representative graphs and statistics of H&E and IHC to verify ANXA8 expression in DGC; b: Immunoblotting assay showing the protein level of ANXA8 and β-actin protein in control (Ctr) and SgANXA8 MKN45 cell lines. c: Immunoblotting assay

showing the protein level of ANXA8 and β-actin protein in control (Ctr) andSgANXA8 SNU-1 cell lines. d and e: Heatmap of differentially expressed proteins between control and SgANXA8 MKN45 cell. Results are presented as mean ± standard deviation in a. n: Biological duplication per group. Student t test, ****P <.0001.

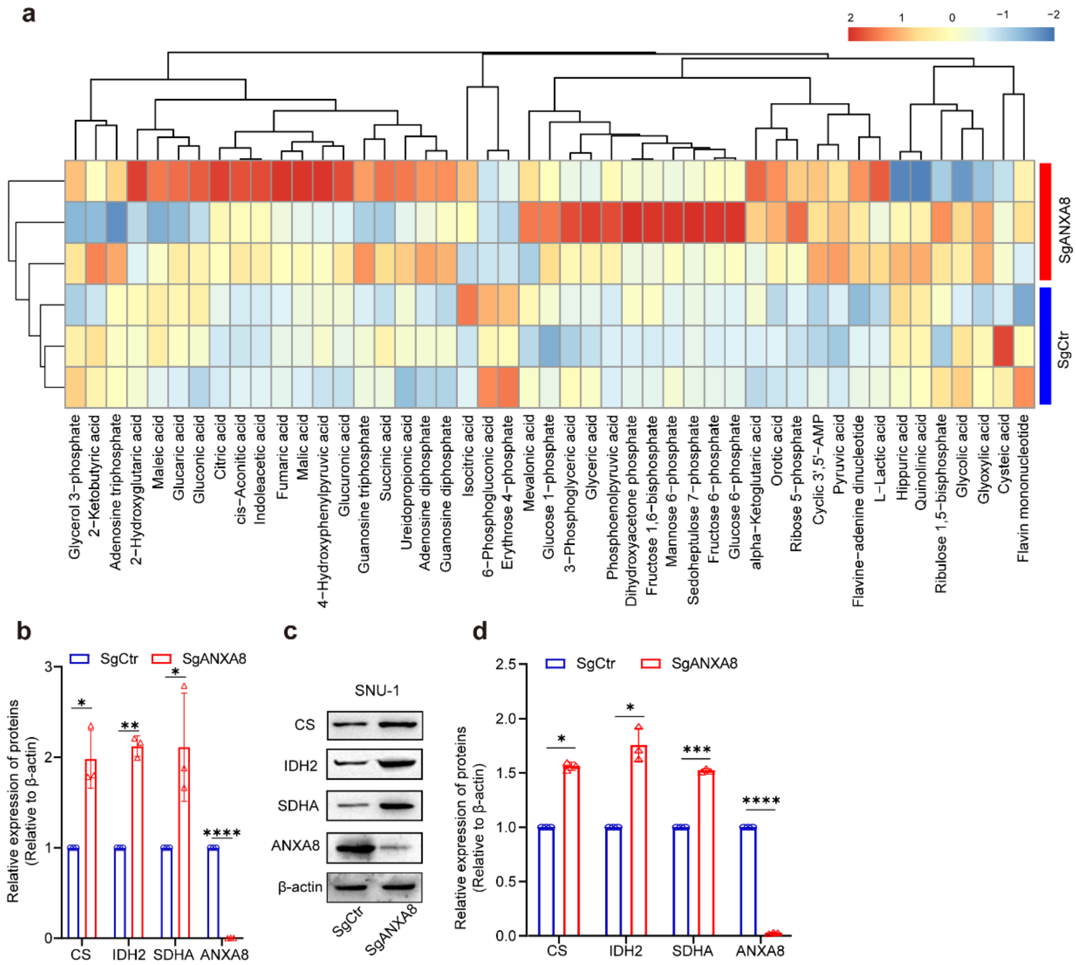

Supplementary Figure 7. Metabolomics analysis and Immunoblotting assay **between control (Ctr) and SgANXA8 MKN45 cells**

a: Heatmap plot showing differentially expressed metabolites between control and SgANXA8 MKN45 cells. b: Immunoblotting assay showing protein levels of CS, IDH2, SDHA, ANXA8, and β-actin in MKN45 cells. c and d: Quantitative statistics and Immunoblotting assay showing protein levels of CS, IDH2, SDHA, ANXA8, and β-actin in SNU-1 cells. Results are presented as mean ± standard deviation in b and d. n: Biological duplication per group. Student t test, *P <.05, **P <.01, ***P <.001, ****P <.0001.

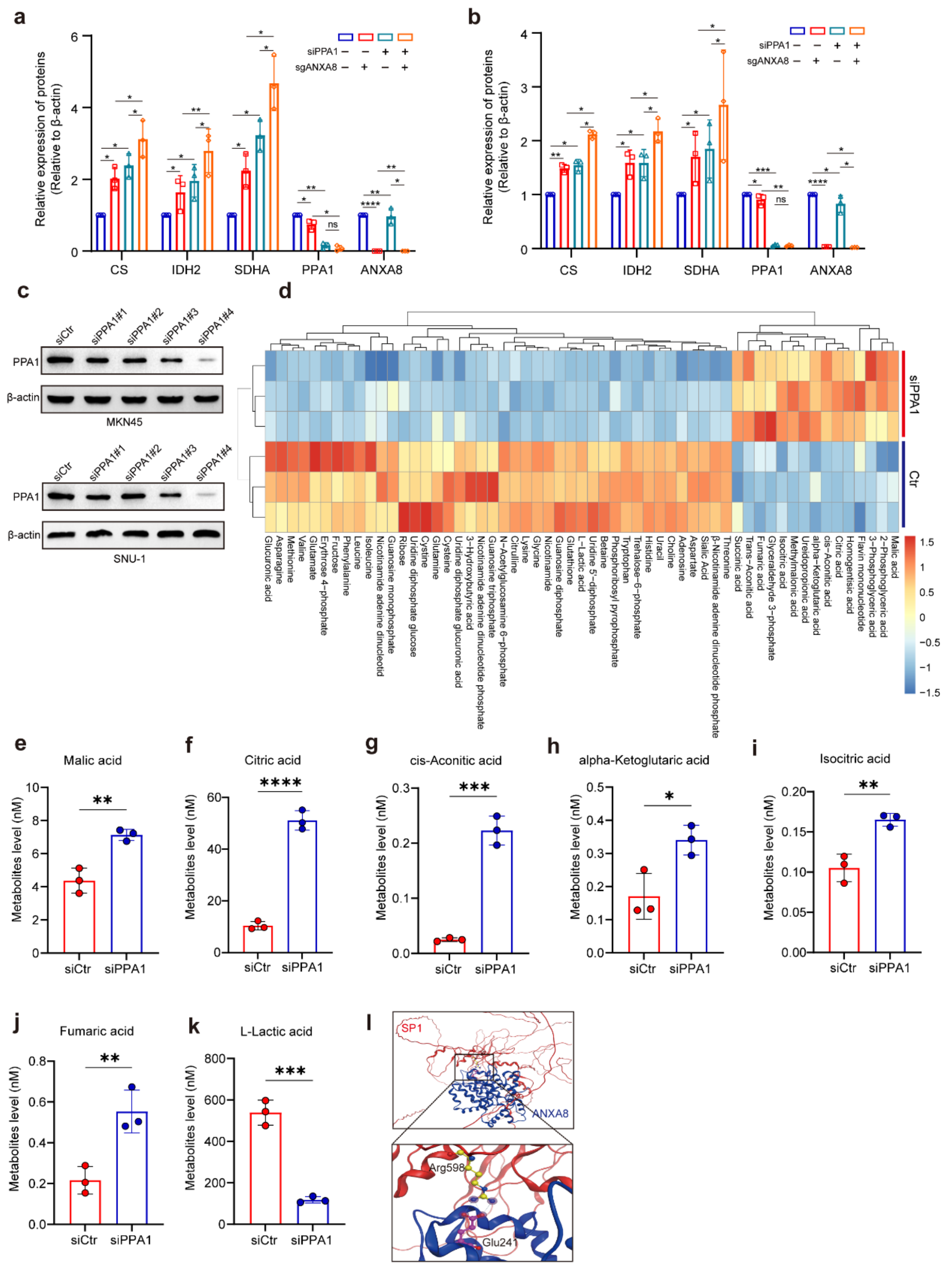

Supplementary Figure 8. ANXA8 Regulates the TCA Cycle and ECM through Interaction with PPA1 and SP1

a and b: Quantitative statistics of immunoblotting assay showing the protein level of CS, IDH2, SDHA, ANXA8,PPA1 and β-actin protein in Ctr and SgANXA8 with or without siPPA1 in MKN45 and SNU-1cells. c:Immunoblotting assay showing the protein level of PPA1 and β-actin protein in Ctr and siPPA1 in MKN45 and SNU-1

cells. d-k: Differentially expressed metabolites between siCtr and siPPA1 MKN45 cells. l: AlphaFold3 prediction of the ANXA8-SP1 interaction site. Results are presented as mean ± standard deviation in a and b, e-k. n: Biological duplication per group. Student t test, *P <.05, **P <.01, ***P <.001, ****P <.0001, ns: not significant.

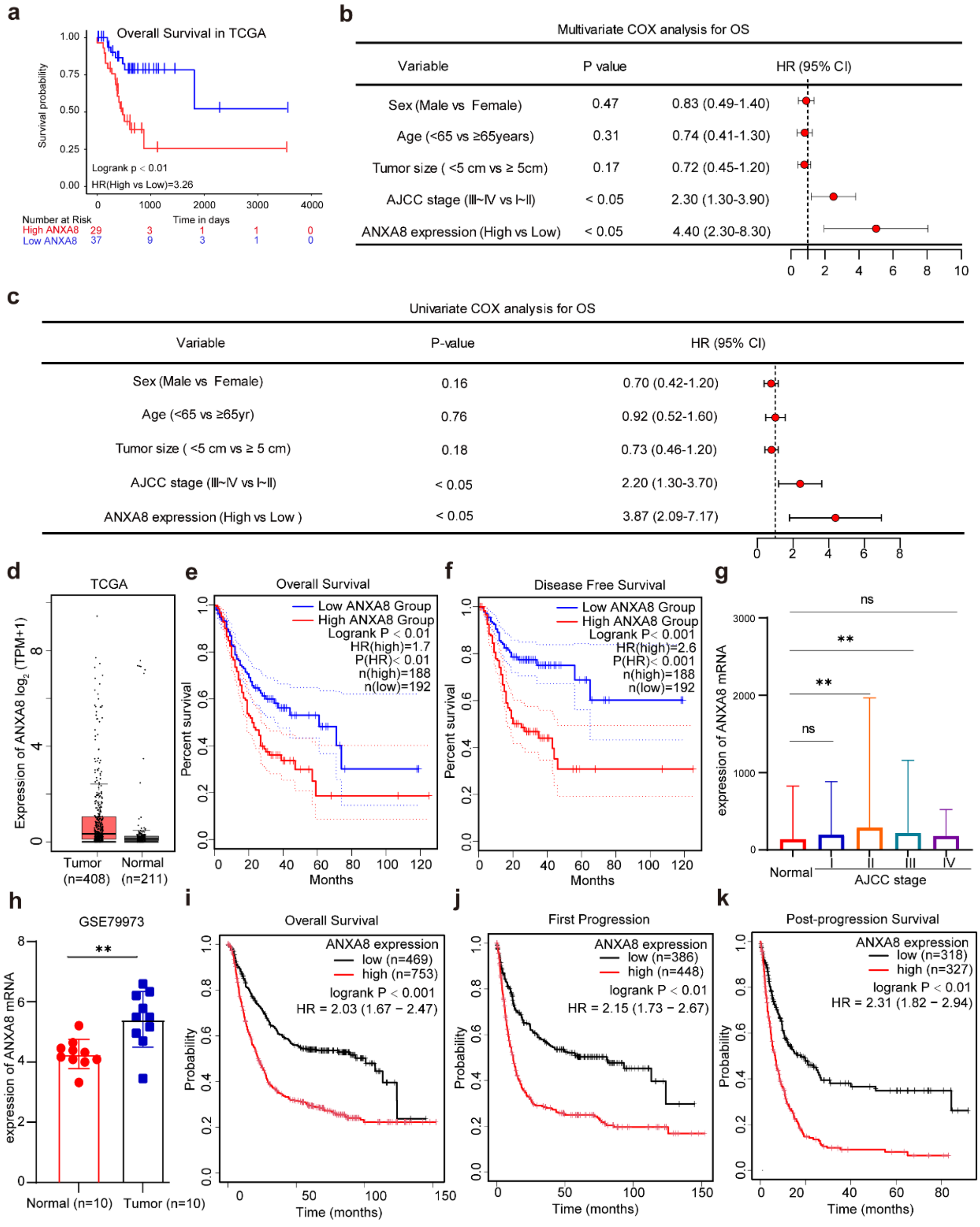

Supplementary Figure 9. Prognostic significance of ANXA8 in GC

a: Kaplan-Meier analysis of the correlation between ANXA8 expression (n = 62) and prognosis of patients with DGC in TCGA. b and c: Cox model results predicting OS in 90 patients with DGC. d: ANXA8 mRNA expression of gastric cancer (GC) in

TCGA data. e and f: Kaplan-Meier analysis of the correlation between ANXA8 expression and prognosis of GC patients in TCGA data. Significance was assessed using the Log-rank (Mantel Cox) test. g: Correlation between ANXA8 and AJCC staging of GC in TCGA data. h: ANXA8 mRNA expression of GC in GEO data. i-k: Kaplan-Meier analysis of the correlation between ANXA8 expression and prognosis of patients with GC in GEO data. Significance was assessed using the Log-rank (Mantel Cox) test. Results are presented as mean ± standard deviation in g and h. n: Biological duplication per group. Student t test, **P <.01, ns: not significant.

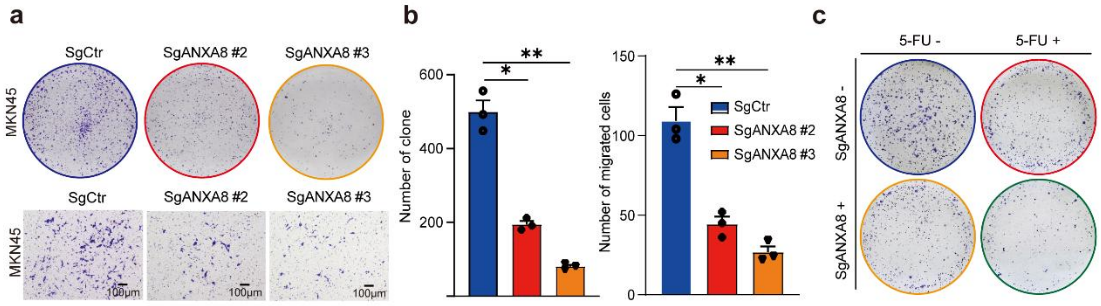

Supplementary Figure 10. Colony Formation Assays Assessing Sensitivity in MKN45 Cell Lines with Variable Treatments and Genetic Modifications

a and b: Representative images and quantification of colony formation assays assessing sensitivity in control and SgANXA8 MKN45 cell lines. c: Representative images of colony formation assays assessing sensitivity in 5-FU and SgANXA8 MKN45 cell lines. Results are presented as mean ± standard deviation in b. n: Biological duplication per group. Student t test, *P <.05, **P <.01.

a

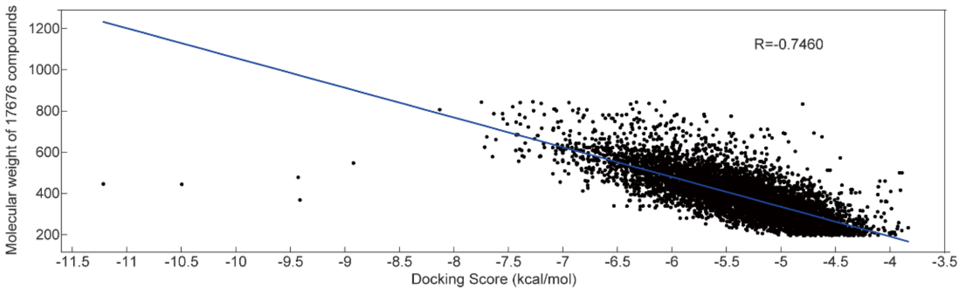

b

	Molecular	Docking Score	Name	CAS	Molecular weight
1	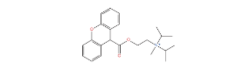	-11.2109	Propantheline bromide	50-34-0	448. 4000
2	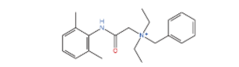	-10.4914	Denatonium benzoate	3734-33-6	446. 5800
3	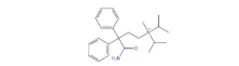	-9.4241	Isopropamide Iodide	71-81-8	480. 4300
4	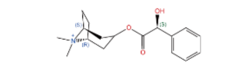	-9.4077	Homatropine Methylbromide	80-49-9	370. 2900
5	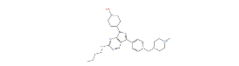	-8.9171	UNC2025	T4419	549. 6200
6	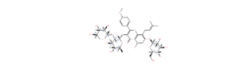	-8.1254	Epimedin B	110623-73-9	808. 7800
7	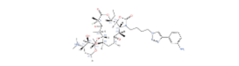	-7.7432	Solithromycin	760981-83-7	845. 0100
8	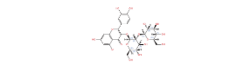	-7.7139	Quercetin-3-O-sophoroside	18609-17-1	626. 5200
9	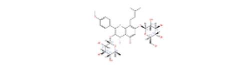	-7.6937	Icariin	489-32-7	676. 6800
10	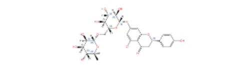	-7.6413	Narirutin	14259-46-2	580. 5300
11	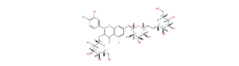	-7.6294	Isoquercitrin-7-O-gentiobioside	60778-02-1	788. 6600
12	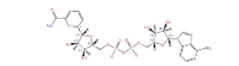	-7.6111	NAD$^+$	53-84-9	663. 4300
13	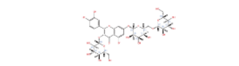	-7.5495	Isoquercitrin-7-O-gentiobioside	60778-02-1	788. 6600
14	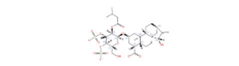	-7.5444	Atractyloside potassium salt	102130-43-8	763. 8800
15	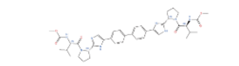	-7.5055	Daclatasvir	1009119-64-5	738. 8800

Supplementary Figure 11. Screening of effective small-molecule inhibitors for ANXA8

a: Affinity results of 17,676 small molecules screened against ANXA8 using virtual screening. b: Characteristics and properties of the 14 potential ANXA8 inhibitors identified.

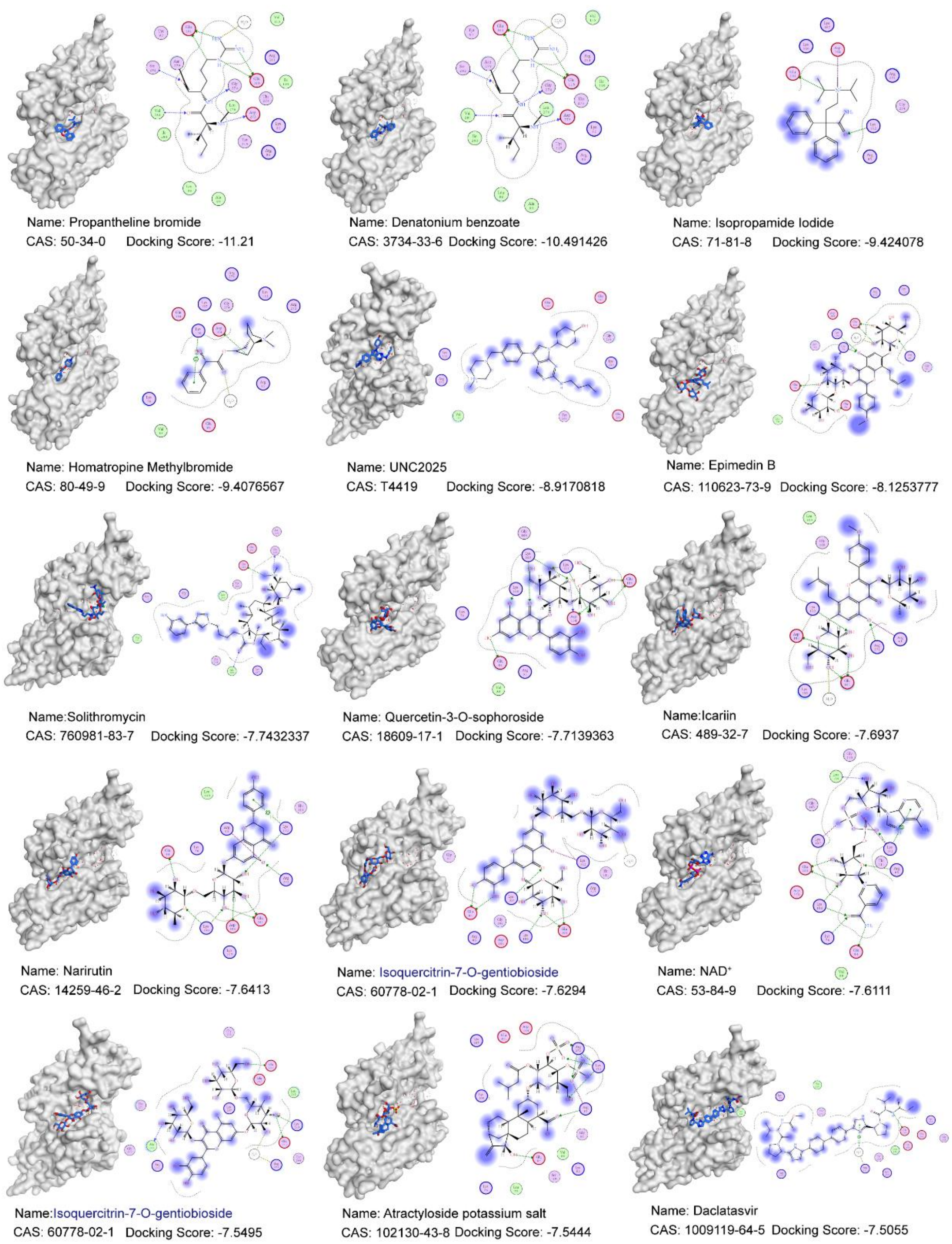

Supplementary Figure 12. Schematic representation of the interaction of ANXA8 protein with 14 small-molecule inhibitors.

Blue color indicates two interactions of the same small molecule inhibitor.

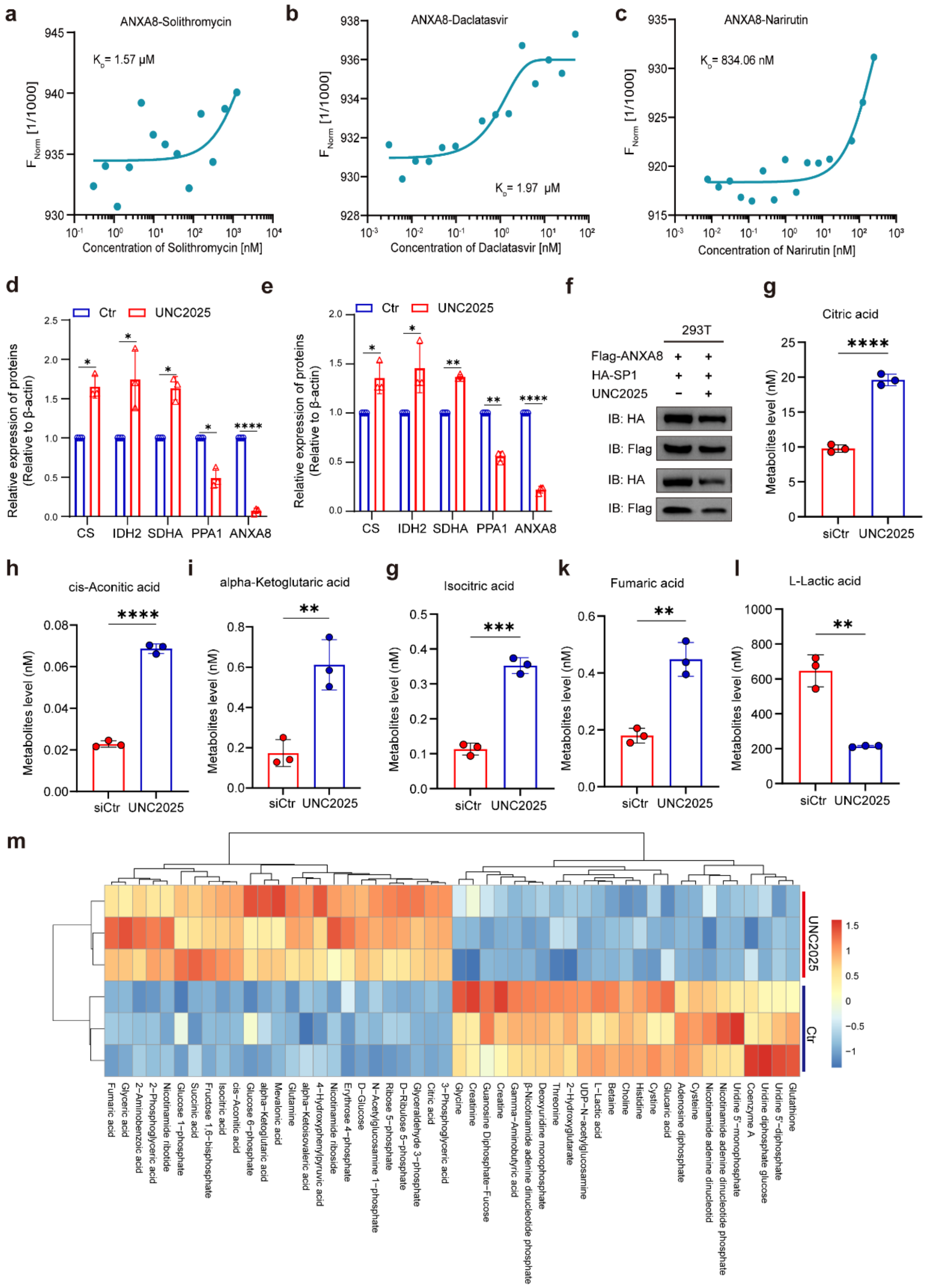

Supplementary Figure 13. Identification and characterization of UNC2025 as an ANXA8 inhibitor

a-c: Kinetic constant (KD) analysis for the interaction of Solithromycin, Daclatasvir, and Narirutin with ANXA8 using MST. d: Quantitative statistics of immunoblotting assay showing the protein level of CS, IDH2, SDHA, ANXA8,PPA1 and β-actin

protein in Ctr and treatment (UNC2025, 5μM) of MKN45 cells. e: Quantitative statistics of immunoblotting assay showing the protein level of CS, IDH2, SDHA, ANXA8,PPA1 and β-actin protein in Ctr and treatment (UNC2025, 5μM) of SNU-1 cells. f: Co-IP assays were employed to assess the interaction of after UNC2025 treatment ANXA8 and SP1 in 293T cells expressing the indicated plasmids, with GFP serving as a control. g-m: Differentially expressed metabolites between Ctr and treatment (UNC2025, 5μM) of MKN45 cells. Results are presented as mean ± standard deviation in d, e and g-l: Biological duplication per group. Student t test, *P <.05, ***P <.001, ****P <.0001.

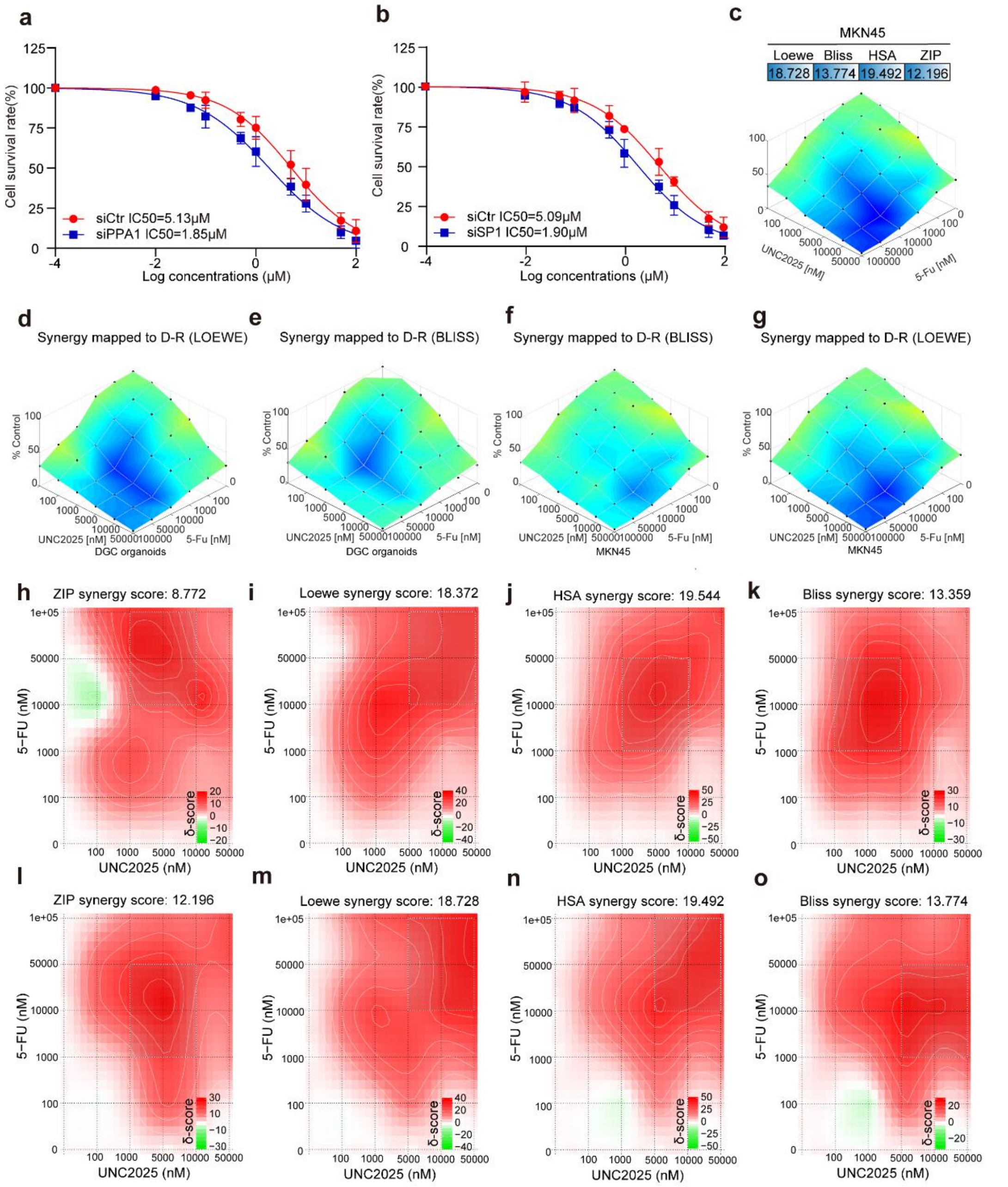

Supplementary Figure 14. Synergistic effect of the small-molecule inhibitor UNC2025 with 5-FU

a: Sensitivity to UNC2025 in siCtr and siPPA1 MKN45 cells (n = 3). b: Sensitivity to UNC2025 in siCtr and siSP1 MKN45 cells (n = 3). c: Synergistic score plots for the combination of UNC2025 and 5-FU in MKN45 cells using the ZIP, Loewe, Bliss, and HSA models; d-g: Synergistic score plots for the combination of UNC2025 and 5-FU in organoids using the ZIP, Loewe, Bliss, and HSA models; h-k: Synergistic score plots for UNC2025 and 5-FU in MKN45 cells using the ZIP, Loewe, Bliss, and HSA

models. l-o: Synergy analysis of the combination of UNC2025 and 5-FU in DGC organoids and MKN45 cells using the Loewe, and Bliss independence models.

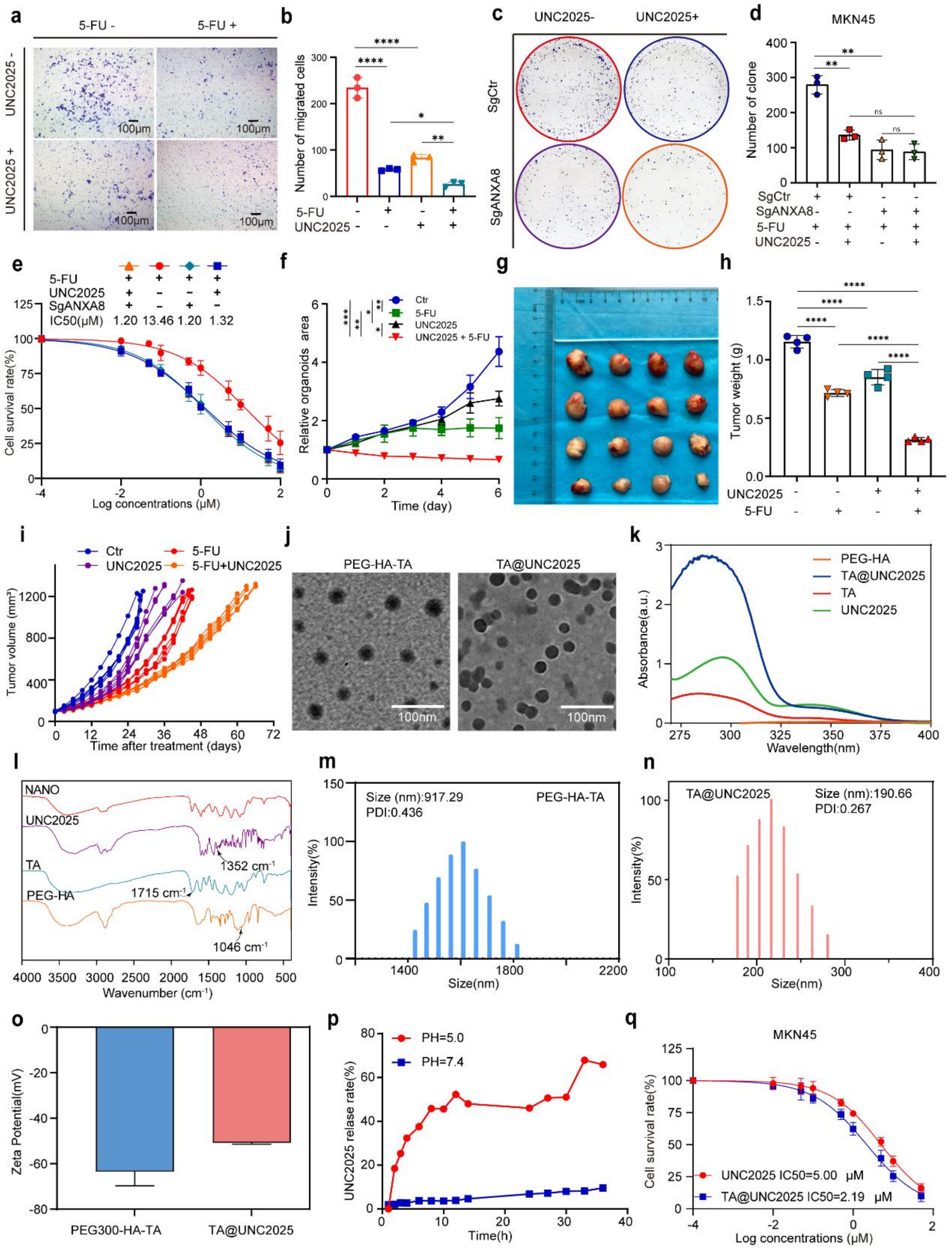

Supplementary Figure 15. Nanopackaging of UNC2025 enhances its synergistic effect with 5-FU and increases 5-FU efficacy

a and b: Representative images and quantification of invasion assays with 5-FU, UNC2025, or both (n =3) in MKN45 cells. c and d: Representative images and quantitative statistics of the effects of UNC2025, SgANXA8, and their combination on

MKN45 cell colony formation in the presence of 5-FU. (n =3). e: Sensitivity of UNC2025, SgANXA8 and their combinations in MKN45 in the presence of 5-FU. (n = 3). f: Growth response of PDOs to UNC2025 (1 μM) and 5-FU (20 μM) (n = 6). g: Raw tumor volume in tumor tissues of PDXs treated with UNC2025 and/or 5-FU. h: Raw tumor weight in PDXs treated with UNC2025, 5-FU, or both (n = 4). i: Tumor growth in PDXs treated with UNC2025, 5-FU, or both (n = 6). j: Transmission electron microscopy was used to characterize the morphology of TA@UNC2025, Scale bar, 100 nm. k: ultraviolet -vis spectrum of PEG300-HA, TA, UNC2025, and TA@UNC2025 in DMSO. l: FT-IR spectra of PEG-HA, TA, UNC2025, and TA@UNC2025. m and n: Hydrodynamic diameter of PEG-HA-TA and TA@UNC2025 examined in pH 7.4 buffer. o: Zeta potential of PEG-HA-TA and TA@UNC2025. p: UNC2025 release profiles from TA@UNC2025 examined in pH 7.4 PBS and pH 5.0 PBS UNC2025 release profiles from TA@UNC2025 examined in pH 7.4 PBS and pH 5.0 PBS. q: Sensitivity to UNC2025 and TA@UNC2025 in MKN45 cells (n = 3). Results are presented as mean ± standard deviation in b, d and h. n: Biological duplication per group. Student t test, *P <.05, **P <.01, ***P <.001, ****P <.0001, ns: not significant. Polydispersity (PDI)

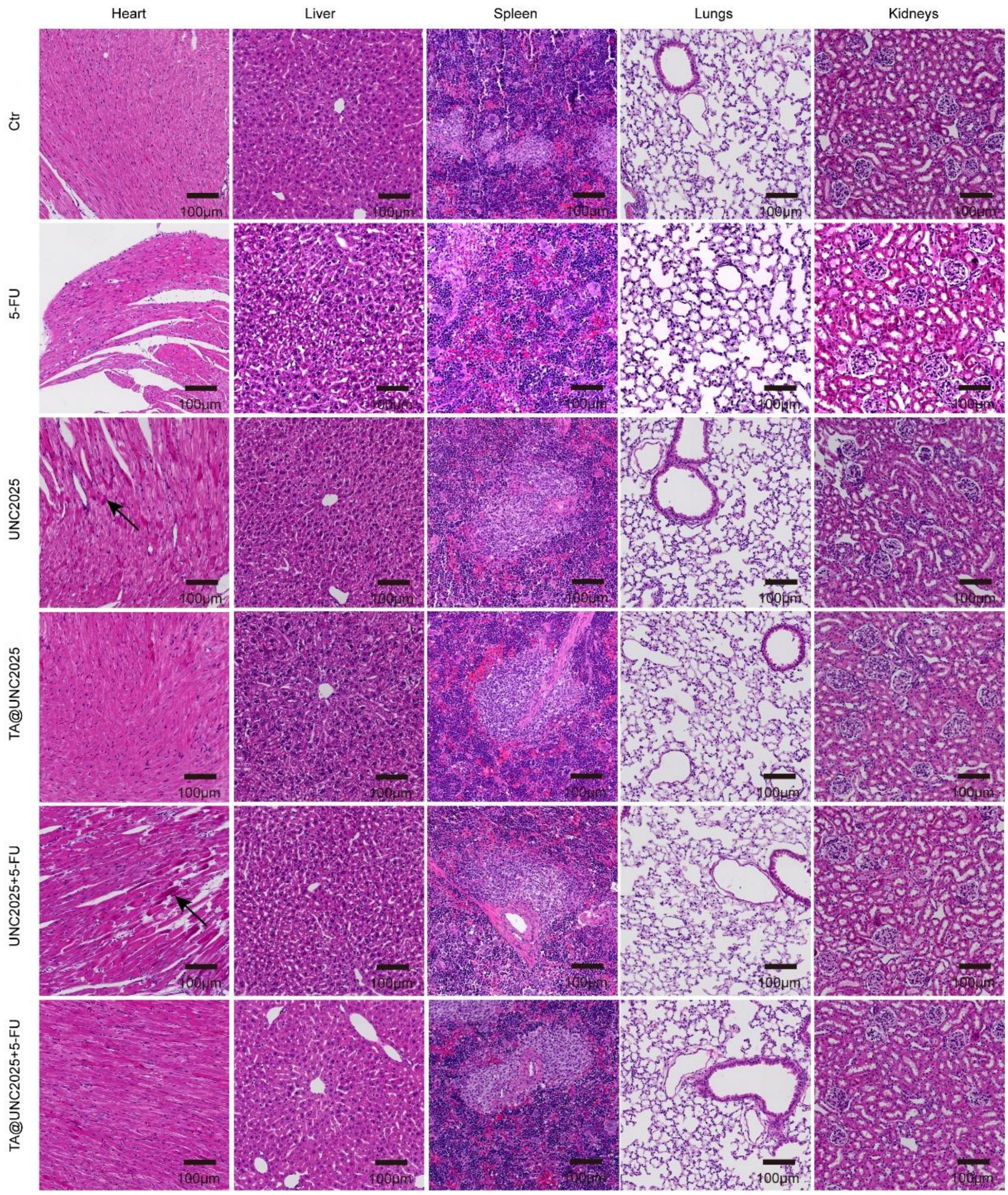

Supplementary Figure16. Assessment of the biological safety of drugs

a: Representative HE staining images of various treatment plans for the assessment of biosafety in the heart, liver, spleen, lungs and kidneys. The black arrow indicates disintegration and fragmentation of myocardial fibers.
